# Genetic heterogeneity in patients with enlarged vestibular aqueduct and Pendred syndrome

**DOI:** 10.1186/s10020-025-01262-x

**Published:** 2025-05-27

**Authors:** Marek Sklenar, Silvia Borecka, Lukas Varga, Emanuele Bernardinelli, Juraj Stanik, Martina Skopkova, Miroslav Sabo, Diana Ugorova, Silvia Dossena, Daniela Gasperikova

**Affiliations:** 1 Diabgene Laboratory, Institute of Experimental Endocrinology, Biomedical Research Center, Slovak Academy of Sciences, Dubravska cesta 9, Bratislava, 845 05 Slovakia; 2 Department of Otorhinolaryngology - Head and Neck Surgery, Faculty of Medicine and University Hospital Bratislava, Comenius University, Bratislava, Slovakia; 3 Institute of Pharmacology and Toxicology, Paracelsus Medical University, Salzburg, 5020 Austria; 4 Department of Paediatrics, Faculty of Medicine, National Institute of Children’s Diseases, Bratislava, Slovakia; 5 Research and Innovation Center Regenerative Medicine & Novel Therapies, Paracelsus Medical University, Salzburg, 5020 Austria

**Keywords:** *SLC26A4*, CEVA haplotype, Enlarged vestibular aqueduct, Goiter, Hearing loss

## Abstract

**Background:**

Pathogenic variants in the *SLC26A4* gene, encoding for Cl^−^/HCO_3_^−^ and I^−^ anion transporter pendrin, are associated with non-syndromic hearing loss with enlarged vestibular aqueduct (NSEVA) and Pendred syndrome (PDS). In the Caucasian population, up to 75% of patients fail to identify a genetic cause through biallelic mutations in the *SLC26A4* gene. The CEVA haplotype could therefore play an important role in the diagnostics of NSEVA. The aim of the study was to determine the genetic etiology of hearing loss with EVA or with fully developed PDS in 37 probands and the functional characterization of novel variants identified in the *SLC26A4* gene.

**Methods:**

To determine the genetic etiology, Sanger sequencing, WES and KASP genotyping assay were used. Functional characterization of *SLC26A4* variants c.140G>A (p.R47Q), c.415G>A (p.G139R), c.441G>A (p.M147I), c.481T>A (p.F161I), c.1589A>C (p.Y530S) and c.2260del (p.D754Ifs*5) involved determination of iodide influx, total and plasma membrane pendrin expression level and subcellular localization of pendrin by confocal imaging. The nanopore sequencing of nasopharyngeal swab samples was performed to confirm the pathogenic effect of potential splice site variant c.415G>A.

**Results:**

Biallelic variants in the *SLC26A4* gene (M2 genotype) were identified in ten probands and a complete CEVA haplotype was confirmed in three probands harbouring *SLC26A4* monoallelic variants (M1 genotype). Fifteen variants in the *SLC26A4* gene were identified in total, three of which are novel. The functional characterization of the novel variants and variants which were not yet functionally characterized confirmed the pathogenic potential of five out of six tested variants (p.G139R, p.M147I, p.Y530S, p.D754Ifs*5, and p.F161I). Analysis of nasopharyngeal swab samples confirmed exon 4 skipping due to novel variant *SLC26A4*:c.415G>A. Probands with biallelic *SLC26A4* variants had significantly larger thyroid volume per m^2^ of body surface area than subjects with monoallelic *SLC26A4* variants and the CEVA haplotype.

**Conclusions:**

The genetic aetiology was determined in 13 out of 37 probands (35%), seven manifested with PDS and six with NSEVA. The present study highlights the importance of functional testing to confirm the pathogenicity of *SLC26A4* variants and the phenotype-genotype correlation in *SLC26A4*-related disorders.

**Supplementary Information:**

The online version contains supplementary material available at 10.1186/s10020-025-01262-x.

## Background

Hearing loss (HL) is one of the most common sensory disorders affecting approximately one to three out of 1000 newborns, with genetic factors being the leading cause (Morton et al. 2006, Korver et al. 2017; Al-Ani 2023). The prevalence of genetic causes varies by ethnicity. Pathogenic variants in the *GJB2* gene, which encodes the connexin 26 protein, are the most frequent genetic cause of HL, accounting for up to 50% of non-syndromic HL in various populations (Kenneson et al. 2002). In Japan, mutations in the *SLC26A4* gene represent the second most common cause of autosomal recessive HL, responsible for 5% of cases (Usami et al. 2022). In contrast, European data indicate that variants in the *STRC* gene are the second most common (16%), while pathogenic variants in the *SLC26A4* gene occur with a frequency of 3.8% (Del Castillo et al. 2022).

The *SLC26A4* gene encodes pendrin, a transmembrane anion exchanger expressed in the inner ear, thyroid and kidney (Roesch et al. 2021). In the cochlea, vestibular labyrinth, and endolymphatic sac, pendrin facilitates Cl⁻/HCO₃⁻ exchange at the apical membrane of epithelial cells, thereby regulating endolymphatic pH and ionic composition essential for both auditory and vestibular function (Royaux et al. 2003; Liu et al. 2023). In the thyroid, pendrin is involved in the transport of iodide into the follicular lumen, a critical step in thyroid hormone biosynthesis (Royaux et al. 2000).

At the cellular level, some *SLC26A4* variants may impair protein folding, leading to retention and degradation of pendrin in the endoplasmic reticulum, or result in reduced protein levels at the plasma membrane despite successful trafficking—both of which compromise pendrin function (Rapp et al. 2017; Bernardinelli et al. 2025).

Pathogenic variants in *SLC26A4* can cause two related autosomal recessive disorders: non-syndromic enlarged vestibular aqueduct (NSEVA; MIM #600791) and Pendred syndrome (PDS; MIM #274600). NSEVA is characterized by hearing loss with inner ear malformations such as an enlarged vestibular aqueduct (EVA) and sac either alone or in conjunction with cochlear incomplete partition type II (Mondini malformation), while PDS presents with EVA, goiter, and variable hypothyroidism (Yang et al. 2005; Ito et al. 2011; Wémeau et al., 2017). The severity of these phenotypes correlates with genotype: PDS typically involves biallelic variants in either the homozygous or compound heterozygous state (M2 and “M2” genotype), whereas NSEVA is frequently associated with monoallelic (M1) or no detectable coding-region variants (M0) in *SLC26A4* (Pryor et al. 2005; Choi et al. 2009a; Ito et al. 2011).

In some M1 NSEVA cases, the missing heritability may be explained by a recently identified Caucasian EVA-associated (CEVA) haplotype, a region located upstream of the gene *SLC26A4.* This haplotype is characterized by two rare single-nucleotide deletions and ten single-nucleotide substitutions in the intergenic regions or introns of genes such as *PIK3CG*, *PRKAR2B*, *HBP1*, *COG5*, *DUS4L* and *BCAP29* (Chattaraj et al. 2017; Smits et al. 2022). Digenic inheritance involving *SLC26A4* and additional genes (*FOXI1*,* KCNJ10*,* EPHA2*) has also been proposed (Yang et al. 2007, 2009; Li et al. 2020), although the roles of *FOXI1* and *KCNJ10* remain controversial (Landa et al. 2013; Zhao et al. 2014, 2019).

In this study, we aimed to elucidate the genetic cause of disease in a cohort of 37 Slovak probands presenting with sensorineural or mixed HL and EVA, including seven individuals with clinical features of PDS. We additionally conducted functional characterization of six *SLC26A4* variants to explore their impact on pendrin function, expression and localization. We also examined the potential pathological effect of the putative missense variant on pre-mRNA splicing by Oxford nanopore sequencing. Our findings provide novel insights into the molecular pathophysiology of *SLC26A4*-related disorders and their phenotypic heterogeneity.

## Methods

### Subjects

The study included 37 Slovak probands of Caucasian ethnicity with sensorineural or mixed hearing loss associated with EVA or PDS, without clinical manifestations of other distinct syndromic diseases. Malformations of the inner ear were assessed using computed tomography (CT) and/or magnetic resonance imaging (MRI) of the temporal bones. The severity of hearing loss was determined based on the results of side-specific pure-tone audiometry, and the type of hearing loss was defined as conductive, sensorineural, or mixed by air- and bone-conduction thresholds. Additional clinical data e.g. on head trauma, progression of hearing loss, presence of goiter, and the onset of hearing loss concerning speech development, defined as congenital, prelingual, perilingual, and postlingual were reviewed from the subjects’ medical records. Subjects with confirmed pathogenic or likely pathogenic variants in the *SLC26A4* gene in homozygous or compound heterozygous state and patients with a single variant in the *SLC26A4* gene in combination with the CEVA haplotype were screened for thyroid parameters. Details on thyroid gland examination are given in the Supplementary Information.

### Genomic DNA analysis

Details on DNA sampling, isolation and DNA analysis using Sanger sequencing and whole-exome sequencing (WES) are given in the Supplementary Information. For the prioritization of variants through WES analysis, a virtual gene panel was used, containing 579 genes associated with hearing loss (Supplementary Table S2).

### Nanopore sequencing

RNA was isolated from nasopharyngeal swabs collected in duplicate from D1215 (compound heterozygote for variants c.1001+1G>A and c.415G>A), his father D2349 (heterozygote for c.415G>A) and a control sample. RNA extraction and cDNA synthesis details are given in the Supplementary Information.

Targeted amplification of the *SLC26A4* transcripts from nasopharyngeal swab-derived cDNA was performed using PCR in a 50 µL reaction volume with primers spanning 538 bp region including exons 2 to 6 of the NM_000441.2 transcript using forward primer complementary to exon 2 (5’ − 3’ sequence: CTT TCC AGC AAC AGC ACG AG) and reverse primer complementary to exon 6 (5’ − 3’ sequence: AAT CCA ATC TGC AAG CCA CC).

The PCR products were subsequently analyzed by electrophoresis on a 1.5% agarose gel to verify their integrity and purified by QIAquick PCR Purification Kit (Qiagen, Hilden, Germany). Concentrations of purified PCR products were measured with the Qubit dsDNA High Sensitivity Assay Kit (Thermo Fisher Scientific, Waltham, MA, USA).

The preparation of amplicon ends for adapter ligation, followed by the ligation of native barcodes and sequencing adapters, as well as library preparation, was performed using Native Barcoding Kit 24 V14 (SQK-NBD114.24) following the manufacturer′s instructions (Oxford Nanopore Technologies, Oxford, England, United Kingdom). The final pooled libraries were sequenced on MinION Mk1C using R10.4.1 flow cells (FLO-MIN114) and MinKNOW v.23.07.5 software. Only passed reads were used for subsequent analyses.

Sequencing data were aligned to both human reference *SLC26A4* transcript (NM_000441.2) and human genome (Ensembl primary assembly GRCh38). Namely, raw barcoded reads were basecalled and demultiplexed using the Dorado v.0.8.3 tool integrated within the MinKNOW package (https://github.com/nanoporetech/dorado/releases/tag/v0.5.0) to generate unaligned BAM files. Alignment to the reference transcript NM_000441.2 sequence was performed via the integrated Minimap2 aligner v.2.28 (Li 2018), and alignments were visualised by Integrative Genomics Viewer (IGV) v.2.11.1 (Robinson et al. 2011). To align sequencing data to the human genome, unaligned BAM files were converted to FASTQ format using Samtools v.1.13. The resulting FASTQ files were then mapped to the reference human genome using the splice-aware alignment tool Minimap2 (Li 2018). Splicing events within the chr7:107,661,600–107,675,150 region were visualised by R package *ggsashimi* v.1.1.5 (Garrido-Martín et al. 2018), applying a filter to exclude splicing events with a frequency below 1.5%.

### Kompetitive allele specific polymerase chain reaction (KASP) assay

All 12 SNPs of the CEVA haplotype (rs17424561, rs79579403, rs17425867, rs117113959, rs17349280, rs117386523, rs80149210, rs199667576, rs9649298, rs117714350, rs199915614, rs150942317) were genotyped by competitive allele-specific PCR with fluorescence detection (KASP assay). KASP Primer mix contained two allele-specific primers designed by the manufacturer with a unique unlabelled tail at the 5′ end and one common reverse primer. KASP Master mix consisted of two fluorescently labelled FRET cassettes, ROX passive reference dye and other components essential for PCR reaction. Reaction mixtures preparation and PCR amplification were performed according to the manufacturer′s protocol (LGC Genomics, Herts, United Kingdom). KASP genotyping was performed on the real-time PCR instrument QuantStudio™ 5 System (LifeTechnologies, Carlsbad, CA, USA).

### Plasmid constructs

For functional characterization of selected variants identified in the *SLC26A4* gene, two types of plasmids were prepared. The pTARGET vector, containing the cDNA of wild-type or mutated human pendrin, was used for functional tests. The pEYFPN1 vector, containing the cDNA of wild-type or mutated human pendrin fused in frame with the ORF of the enhanced yellow fluorescent protein (EYFP), was used for co-localization and determination of pendrin expression levels by confocal imaging (de Moraes et al. 2016). The pendrin variants were produced using the QuikChange Lightning Site-Directed Mutagenesis Kit (Agilent Technologies, Santa Clara, CA, USA) according to the manufacturer’s protocol, using the primers listed in Supplementary Table S3. The sequence of all plasmid inserts was verified by Sanger sequencing before use in experiments.

Moreover, a plasmid encoding the iodide-sensitive EYFP variant H148Q;I152L (Galietta et al. 2001) was used for the functional test and pECFPC vector (Clontech, Mountain View, CA, USA) bearing the transfection marker enhanced cyan fluorescent protein (ECFP) was used for determination of expression level in the plasma membrane by confocal imaging.

### Cell lines and transient transfection

Derivative of Human embryonic kidney (HEK) 293 cells - Phoenix (DiCiommo et al. 2004) and the HeLa cell line (human cervical adenocarcinoma, CCL-2, acquired from American Type Cell Culture Collection (ATCC), Manassas, VA, USA) were cultivated in Minimum Essential Eagle Medium (Sigma-Aldrich, St. Louis, MO, USA) with the supplementation with 10% fetal bovine serum (GIBCO, Thermo Fisher, Waltham, MA, USA), 2 mM L-glutamine, 100 U/mL penicillin, 100 mg/mL streptomycin, and 1 mM pyruvic acid (sodium salt). Cells were incubated at 37 °C, 5% CO_2_, and 100% humidity.

For functional tests, HEK 293 Phoenix cells were seeded into black 96-well plates, grown overnight until they reached approximately 50% confluence, and co-transfected with 0.12 µg/well of a pTARGET vector containing the cDNA of wild-type or mutated pendrin and 0.12 µg/well of a plasmid encoding the iodide-sensitive EYFP variant H148Q; I152L (Galietta et al. 2001) by the calcium phosphate co-precipitation method (Graham et al. 1973). Functional tests were performed 48 h after transfection at room temperature.

For confocal imaging, HeLa cells were seeded into 6-well plates, grown overnight until they reached approximately 50% confluence, and transfected with 1.5 µg of plasmid DNA/well using METAFECTENE PRO^®^ (Biontex, Munich, Germany), following the manufacturer’s instructions. Afterwards, cells were transferred onto glass slides for microscopy at 48 h post-transfection and imaged at 72 h post-transfection.

### Functional characterization of pendrin variants

Selected pendrin variants were functionally characterized through a series of tests, which included the pendrin functional test (measuring the iodide influx in cells expressing pendrin by fluorometric analysis), determination of pendrin total expression level and expression level in the plasma membrane and co-localization experiments by confocal imaging. Detailed methodological procedures of these tests are provided in the Supplementary Information.

### SLC26A4 modeling

Model of SLC26A4 dimer in complex with Cl^−^ was created using Alphaphold 3 available through alphapholdserver.com (Abramson et al. 2024). For visualisation of variants, mutations were introduced into the WT protein sequence and modelled using ColabFold v1.5.5: AlphaFold2 using MMseqs2 with option using SLC26A4 AlphaFold model available through Uniprot (AF-O43511-F1-model_v4.pdb) as a template (Mirdita et al. 2022). ConSurf scores were counted using the AF-O43511-F1-model_v4.pdb and the ConSurf server (Landau et al. 2005; Ashkenazy et al. 2016). Molecular graphics and analyses were performed with UCSF ChimeraX v1.10 (Meng at al. 2023).

### Statistical analysis

All data are presented as arithmetic means ± standard error of the mean (SEM). Statistical analyses were performed using GraphPad Prism software (version 8.4.3, GraphPad Software, CA, USA). Group differences were assessed using one-way analysis of variance followed by Bonferroni´s or Dunnett´s post-hoc tests for multiple comparisons. Differences between the two groups for the thyroid volume were tested using the Mann-Whitney U test because of non-normally distributed metric data. Statistical significance was defined as *p* < 0.05.

## Results

### Identification of variants in the *SLC26A4* gene and CEVA haplotype

This study involved 37 Slovak probands (19 females and 18 males, aged between 8 and 51 years; median age 19 years, average age 24 years) diagnosed with unilateral or bilateral EVA and bilateral sensorineural or mixed HL. By analyzing the sequence of the *SLC26A4* gene and determining the presence of the CEVA haplotype, we established the genetic aetiology of NSEVA or PDS in 13 probands (35%). Namely, a homozygous state of a pathogenic variant in the *SLC26A4* gene (M2 genotype) was observed in two probands with PDS (D632, D642) and one proband with NSEVA (D930). We identified likely pathogenic and pathogenic variants in the *SLC26A4* gene in a compound heterozygous state (“M2”) in five probands with PDS (D92, D253, D534, D743, D1450) and two probands with NSEVA (D1215, D2128). In three additional probands diagnosed with NSEVA harbouring a monoallelic pathogenic variant in the *SLC26A4* gene (M1 genotype), we identified the complete heterozygous CEVA haplotype with a length of 12 SNPs (D900, D1827, D1966) (Table 1).

**Table 1:** Genotypes and clinical phenotypes of probands with identified variants in the *SLC26A4* gene

Proband ID	Variant Allele 1		Variant Allele 2		CEVA haplotype	Hearing loss phenotype			EVA	Goiter
	Nucleotide change	Amino acid change	Nucleotide change	Amino acid change		Degree	Age at onset	Progression		
**Biallelic** ***SLC26A4*** **cases (M2 and “M2” genotype)**										
D92^a^	c.481T>A	p.F161I	c.1001+1G>A	p.?	GTTCATG-GC-C	Pro	Congenital	Stable	bilat.	Yes
D253^a^	c.1589A>C	p.Y530S	c.1246A>C	p.T416P		L: Mi, R: Pro	Prelingual	Progressive	bilat.	Yes
D534^a^	c.1001+1G>A	p.?	c.1334T>G	p.L445W		Pro	Congenital	Progressive	bilat.	Yes
D632	c.2089+1G>A	p.?	c.2089+1G>A	p.?		L: S, R: Pro	Prelingual	Progressive	bilat.	Yes
D642	c.2089+1G>A	p.?	c.2089+1G>A	p.?		Pro	Congenital	Stable	bilat.	Yes
D743	c.1226G>A	p.R409H	c.2089+1G>A	p.?		L: Mo, R: MoS	Postlingual	Progressive (HT)	bilat.	Yes
D930	c.2089+1G>A	p.?	c.2089+ 1G>A	p.?		L: MoS, R: Pro	Congenital	Stable	bilat.	No
D1215^a^	c.415G>A^b^	p.G139R; p.M103_G139del	c.1001+1G>A	p.?		Pro	Congenital	Progressive	bilat.	No
D1450^a^	c.707T>C	p.L236P	c.890del	p.P297Qfs*6		Pro	Postlingual	Progressive (HT)	bilat.	Yes
D2128^a^	c.1198del	p.C400Vfs*32	c.1226G>A	p.R409H		Pro	Congenital	Progressive	bilat.	No
**Monoallelic** ***SLC26A4*** **cases (M1 genotype) with complete CEVA haplotype**										
D900^a^	c.1001+1G>A	p.?			ACACATG-GC-C	MoS; mixed	Prelingual	Progressive	bilat.	No
D1827^a^	c.2089+1G>A	p.?			ACACATG-GC-C	L: Mo, R: MoS	Prelingual	Stable	bilat.	No
D1966^a^	c.2089+1G>A	p.?			ACACATG-GC-C	Pro	Prelingual	Progressive	bilat.	No
**Monoallelic** ***SLC26A4*** **cases (M1 genotype) with partial CEVA haplotype and** ***KCNJ10*** **variant**										
D619^a^	c.2260del	p.D754Ifs*5			ACATGCATATTA	L: Pro, R: MoS; mixed	Postlingual	Progressive	bilat.	No
**Monoallelic** ***SLC26A4*** **cases (M1 genotype) without CEVA haplotype**										
D533	c.415G>A^b^	p.G139R; p.M103_G139del				Pro	Prelingual	Progressive (HT)	bilat.	No
D1319	c.2089+1G>A	p.?				L: Mi, R: S (15y)	Post (4y)	Progressive (HT)	right	N/A
D1547	c.1790T>C	p.L597S				L: Mo, R: S	Congenital	Stable	right	No
D1560	c.1790T>C	p.L597S				Mi	Congenital	Stable	bilat.	No
D1888	c.441G>A	p.M147I				L: Mi, R: MoS	L: 3y, R: Congenital	Stable	right	No
***SLC26A4 *** **cases with likely benign/benign variants**										
D1998	c.2326C>T	p.R776C				L: MoS, R: Mo; mixed	Congenital	N/A	bilat.	No

*HT* progression of hearing loss after head trauma, *L* left ear, *R* right ear, *Mi* mild, *Mo* moderate, *MoS* moderate-severe, *S* severe, *Pro* profound; *N/A* not available

^a^the *trans* configuration of two recessive variants in the gene *SLC26A4* or the CEVA haplotype was confirmed through cosegregation analysis. The SNPs of the CEVA haplotype (rs17424561, rs79579403, rs17425867, rs117113959, rs17349280, rs117386523, rs80149210, rs199667576, rs9649298, rs117714350, rs199915614, rs150942317) that were detected in each proband are underlined

^b^the variant c.415G>A was predicted to result in an amino acid substitution p.G139R or impact splicing resulting to a truncated protein (p.M103_G139del) before functional studies

**Fig. 1:**
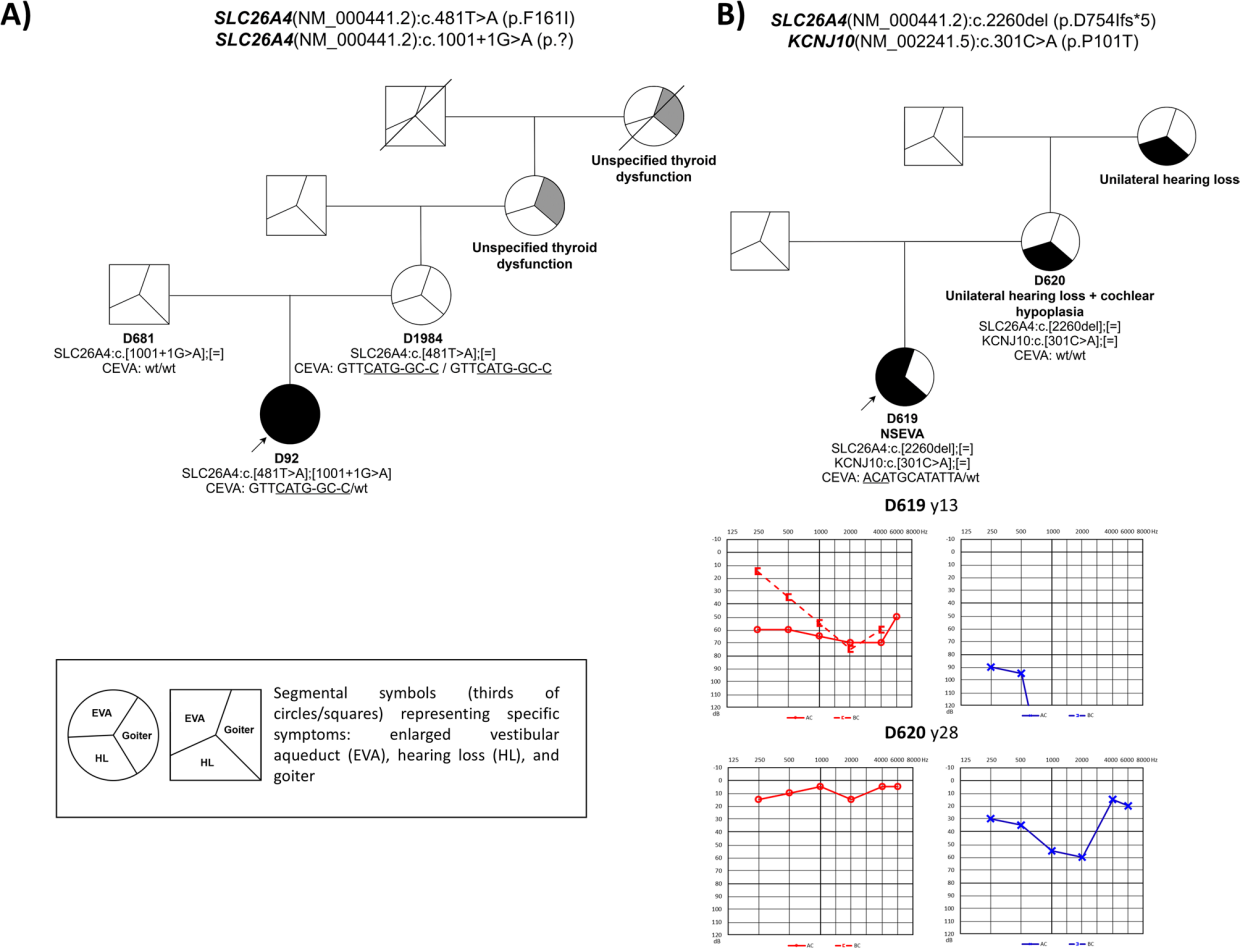
Segregation analysis of the variants in the genes *SLC26A4* and *KCNJ10* and incomplete CEVA haplotype.** A** Pedigree analysis of proband D92 illustrates the inheritance of variants c.1000+1G>A (p.?) and c.481T>A (p.F161I) in addition to a CEVA haplotype consisting of nine SNPs. The presence of an incomplete homozygous CEVA haplotype alongside a heterozygous hypofunctional p.F161I variant in the unaffected mother (D1984) raises questions about a causal link between the incomplete CEVA haplotype and NSEVA. **B** Pedigree and pure tone audiometry data for proband D619 and her mother D620 are shown. Both share the variant c.2260del (p.D754Ifs*5) in *SLC26A4* and c.301C>A in *KCNJ10*, with the mother presenting unilateral hearing loss and ipsilateral cochlear hypoplasia without EVA. Proband D619 carries a partial CEVA haplotype consisting of three SNPs

A partial CEVA encompassing nine SNPs (rs117113959, rs17349280, rs117386523, rs80149210, rs199667576, rs9649298, rs117714350, rs199915614, rs150942317) was identified in the family of a proband with PDS (D92). This partial CEVA was identified along with two variants in the *SLC26A4* gene (a likely pathogenic variant c.481T>A and a pathogenic variant c.1001+1G>A). Cosegregation analysis in the family confirmed the *trans* configuration of the variants in the *SLC26A4* gene. The partial nine SNPs CEVA haplotype in a homozygous state, along with the variant c.481T>A, was identified in the normal-hearing mother, which excludes its involvement in the development of hearing impairment. The father is a normal-hearing carrier of the c.1001+1G>A variant (Fig. 1A).

A monoallelic variant in the *SLC26A4* gene with three SNPs of the CEVA haplotype was identified in one proband with NSEVA (D619; Table 1). A monoallelic variant in the *SLC26A4* gene without the CEVA haplotype was identified in five probands with EVA (Table 1).

We did not detect any variants affecting splice sites or coding regions in the *SLC26A4* gene in the remaining 18 probands (in one proband likely benign variant p.R776C was identified).

To further investigate the genetic aetiology of hearing loss we performed the WES analysis in all probands with M1 genotype. Initially, we explored a possible digenic inheritance with variants in *EPHA2*, *FOXI1* and *KCNJ10* as a potential explanation for the missing heritability. One variant of unknown significance (VUS) in the *KCNJ10* gene (NM_002241.5:c.301C>A; p.P101T) in a heterozygous state, along with a novel likely pathogenic variant c.2260del in the *SLC26A4* gene in a heterozygous state and partial CEVA (3 SNPs, rs17424561, rs79579403, rs17425867), was found in proband D619. Cosegregation analysis in the family confirmed the presence of the same variants in the *KCNJ10* and *SLC26A4* genes in the sample from the mother, who was diagnosed with unilateral hearing loss along with ipsilateral cochlear hypoplasia but no EVA. Partial CEVA was not identified in the mother (Fig. 1B).

Analyzing the *EPHA2* gene, we identified the variant NM_004431.5:c.2904G>C (p.Q968H) in a heterozygous state in proband D1319. This variant is predicted to be benign by in silico prediction tools (REVEL = 0.178); it has a relatively high AF of 0.28% (gnomAD v4.1.0) and is classified as likely benign according to the ACMG classification guidelines. This variant was found alongside a pathogenic variant in the *SLC26A4* gene (c.2089+1G>A) in a heterozygous state. No variants were found in the *FOXI1* gene.

Re-analyses of the whole-exome sequencing data in the M1 probands revealed additional variants with unknown significance in additional 13 genes (Supplementary Table S4). None of these variants were assessed as causal for hearing loss phenotype.

### Characterization of identified pendrin variants

**Fig. 2:**
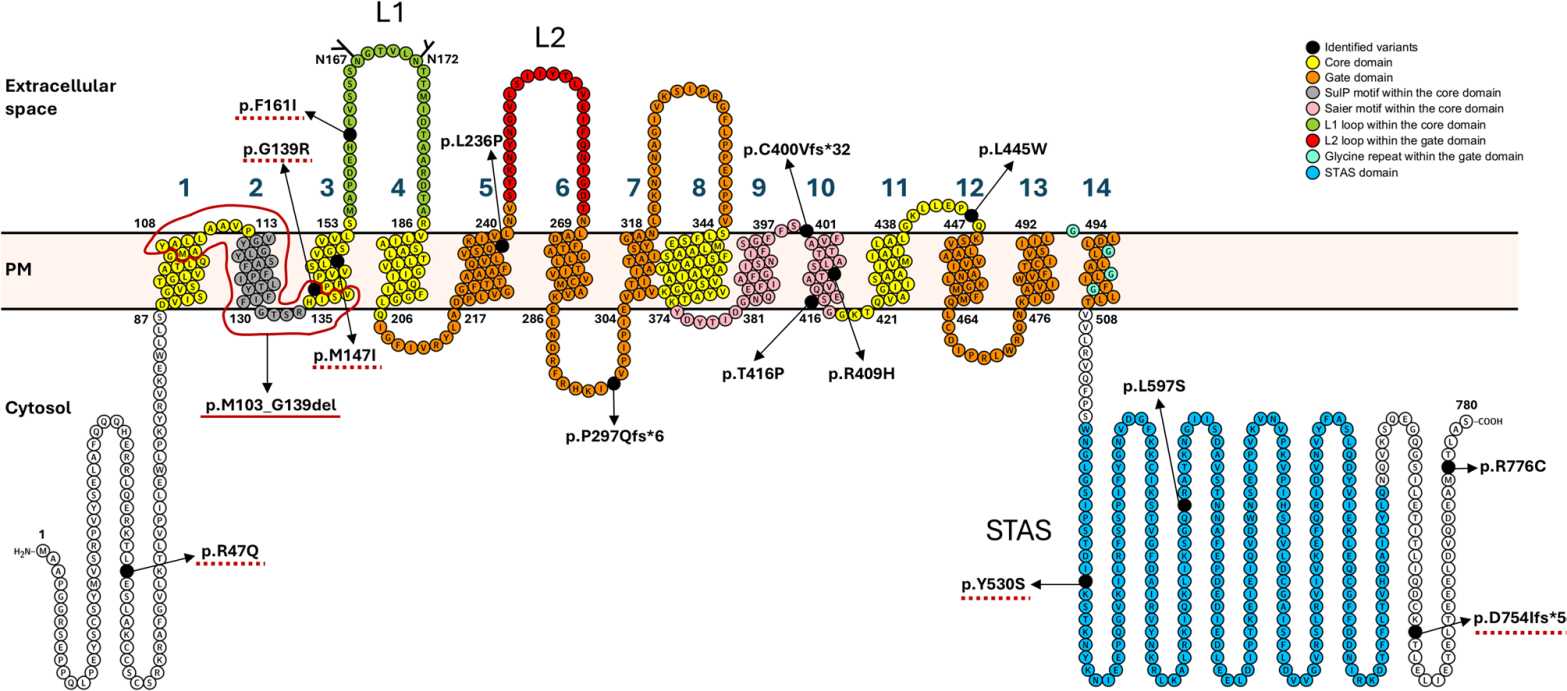
Identified variants mapped onto the topological model of the pendrin protein. Variants identified in this study are marked with black circles, indicating the affected amino acid residues. Variants selected for functional characterization are underlined with a red dashed line. The red solid line highlights the amino acids deleted in the in-frame loss p.M103_G139del. The topology of distinct domains and N-glycosylation sites (N167 and N172) in pendrin model was created using the Protter tool (Omasits et al. 2014) and based on Saier et al. 1999; Zheng et al. 2000; Dossena et al. 2009; Bassot et al. 2017; Rapp et al. 2017; Kuwabara et al. 2018

Fifteen variants in the *SLC26A4* gene were identified in total (Table 2) in patients with EVA, two of which are novel; i.e., they are not yet present in available deafness databases or published literature (p.G139R, p.D754Ifs*5). Two splice-site mutations (c.1001+1G>A and c.2089+1G>A), nine missense variants (p.L236P, p.R409H, p.T416P, p.L445W, p.L597S, p.M147I, p.F161I, p.Y530S, p.R776C) and two frameshift variants (p.P297Qfs*6 and p.C400Vfs*32) have already been identified and documented in NSEVA/PDS patients. The position of individual SLC26A4 variants, considering their location within specific pendrin domains, is illustrated in Fig. 2.

For functional characterization we selected 6 variants (p.M147I, p.F161I, p.Y530S, p.R47Q, p.G139R and p.D754Ifs*5). Three variants have not yet been functionally characterized (p.M147I, p.F161I, and p.Y530S), but have been previously identified in individuals with NSEVA/PDS. Two novel variants (p.G139R and p.D754Ifs*5) were identified in patients with NSEVA. The last novel p.R47Q variant added for functional characterization was previously found in a patient with postlingual moderate progressive hearing loss without EVA and with an identified pathogenic variant c.5668C>T (p.R1890C) in the *TECTA* gene (NM_005422.4) (Pavlenkova et al. 2021).

### Transport function of pendrin variants

**Fig. 3:**
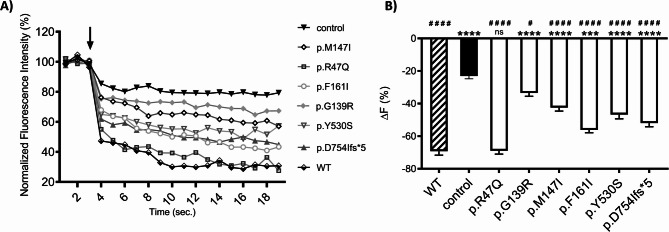
Iodide transport efficiency of wild-type pendrin and six pendrin variants. Pendrin function was determined after co-transfection of HEK 293 Phoenix cells with the wild-type (WT) or pendrin variant and the iodide sensor-enhanced yellow fluorescent protein (EYFP) H148Q;I152L or EYFP H148Q;I152L alone (control). **A** Representative changes in the intracellular fluorescence intensity normalized for the average fluorescence measured before injection of the iodide-containing solution, reflecting pendrin transport efficiency. The arrow indicates the addition of iodide to the extracellular solution. **B** Percentage of intracellular fluorescence decrease over the experimental period (19 s) determined in cells expressing the WT pendrin or the selected pendrin variants and in control cells. *n* = 36 measurements from six independent biological replicates, **** *p* < 0.0001, *** *p* < 0.001, ns: not statistically significant compared to WT; #### *p* < 0.0001, # *p* < 0.05 compared to control, one-way ANOVA with Bonferroni’s multiple comparison post-test

The impact of six pendrin variants on protein function was determined based on the ability to transport iodide anions after heterologous expression in HEK 293 Phoenix cells (Fig. 3).

The novel pendrin variant p.R47Q, identified in a proband with moderate hearing loss without EVA, showed an intact transport function comparable to the wild-type pendrin. Four missense variants (p.G139R, p.M147I, p.F161I, p.Y530S) and one frameshift variant (p.D754Ifs*5) showed a significantly reduced iodide transport capacity compared to wild-type pendrin. On the other hand, these variants retained partial transport activity compared to control cells not expressing pendrin. Among the studied hypofunctional variants, variant p.G139R demonstrated the lowest transport activity, while variant p.F161I demonstrated the highest degree of preserved iodide transport activity. The findings also indicate that the truncated pendrin protein (p.D754Ifs*5) retains partial transport functionality, suggesting the affected protein region at the C-terminus is crucial for achieving optimal transport activity.

### Subcellular localization of pendrin variants

**Fig. 4:**
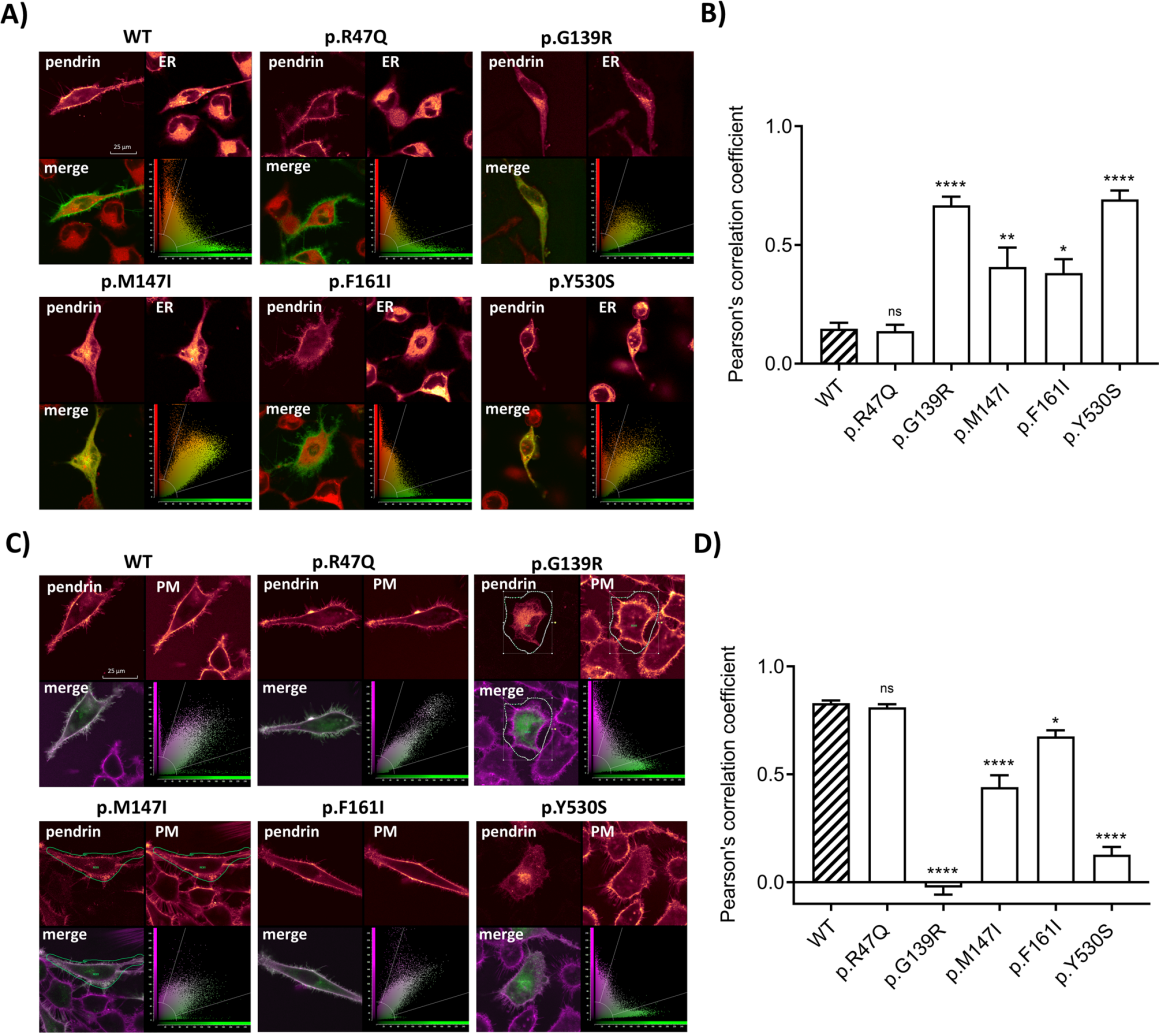
Subcellular localization of wild-type pendrin and five pendrin variants. Co-localization of pendrin protein with the endoplasmic reticulum (ER) and plasma membrane (PM) was determined in living HeLa cells 72 h after transfection with wild-type (WT) or indicated pendrin variant with EYFP fused to the C-terminus and staining with the ERTracker^™^ Red or CellMask^™^ Deep Red plasma membrane stain, respectively. **A** Fluorescent signal of wild-type (WT) or mutant SLC26A4-EYFP (top left), ER (top right), corresponding merged image (bottom left) and scatter plot (bottom right). Scale bar: 25 μm. **B** Pearson’s correlation coefficient indicated the co-localization of WT pendrin and the studied pendrin variants with the ER. **C** Fluorescent signal of WT or mutant SLC26A4-EYFP (top left), PM (top right), the corresponding merged image (bottom left) and scatter plot (bottom right). Scale bar: 25 μm. **D** Pearson’s correlation coefficient indicated the co-localization of WT pendrin and the studied pendrin variants with the PM. **** *p* < 0.0001, ** *p* < 0.01 * *p* < 0.05, ns.: not statistically significant compared to WT, one-way ANOVA with Dunnett’s multiple comparison post-test

The impact of pendrin variants on subcellular localization was determined by defining their co-localization with the plasma membrane (PM) and endoplasmic reticulum (ER) by confocal microscopy, following the overexpression of the variants fused to EYFP in HeLa cells (Fig. 4).

As shown in Fig. 4, wild-type (WT) pendrin is predominantly localized in the PM (Fig. 4C and D), with minimal co-localization with the ER (Fig. 4A and B). The p.R47Q variant exhibited localization at the PM comparable to the wild-type protein, with minimal retention in the ER. On the other hand, variants p.G139R and p.Y530S showed massive retention in the ER, accompanied by a significantly impaired ability to target the PM (*p* < 0.0001). Variants p.M147I and p.F161I co-localized with both the PM and the ER, with significant retention in the ER (*p* < 0.01 and *p* < 0.05, respectively).

### Total and plasma membrane expression levels of pendrin variants

**Fig. 5:**
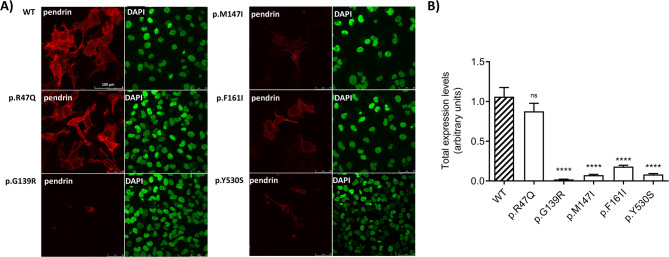
Total protein expression levels of wild-type pendrin and five pendrin variants. HeLa cells were transfected with wild-type (WT) pendrin or the selected variants with EYFP fused to the C-terminus and their expression levels were determined after 72 h using confocal imaging. **A** Images of fixed HeLa cells showing EYFP signal intensity (red, left panels), corresponding to the expression level of pendrin variants and nuclei counterstained with 4’,6-diamidino-2-phenylindole (DAPI, green, right panels). Scale bar: 100 μm. **B** Expression levels of WT pendrin and selected variants expressed as EYFP fluorescence intensity normalized for the cell density. *n* = 24 imaging fields from four biological replicates. **** *p* < 0.0001, ns.: not statistically significant compared to WT, one-way ANOVA with Bonferroni’s multiple comparison post-test

**Fig. 6:**
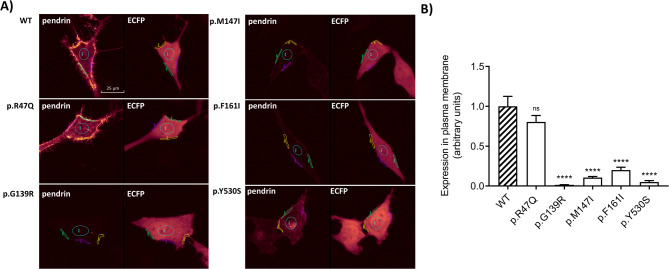
Expression level of wild-type pendrin and five pendrin variants in the plasma membrane region. HeLa cells were cotransfected with wild-type (WT) pendrin or the selected variants with EYFP fused to the C-terminus, and transfection marker-enhanced fluorescent protein (ECFP) and their expression levels in plasma membrane (PM) region were determined in living HeLa cells, after staining with the CellMask^™^ Deep Red plasma membrane stain, 72 h post-transfection. **A** Representative images of the indicated SLC26A4-EYFP variants (left panels) and ECFP fluorescent signal (right panels). Scale bar: 25 μm. **B** Fluorescence intensity of WT and pendrin variants in three regions of the single cell’s plasma membrane (shown in A), averaged and normalized for the fluorescence intensity of ECFP in the cytosol of the same cell. *n* = 12 cells from two biological replicates. **** *p* <0.0001, ns.: not statistically significant compared to WT, one-way ANOVA with Dunnett’s multiple comparison post-test

The total expression of pendrin variants (Fig. 5) and their expression level in the PM (Fig. 6) were monitored using confocal microscopy following heterologous expression in HeLa cells. The total expression level as well as PM expression level of the p.R47Q pendrin variant, which previous analyses confirmed to have an intact transport function and localization in the PM, were similar to that of wild-type pendrin. In contrast, the other four variants (p.G139R, p.M147I, p.F161I, p.Y530S) showed significantly reduced levels of both total pendrin expression and PM expression (*p* < 0.0001).

### Effect of variant c.415G>A on aberrant splicing

**Fig. 7:**
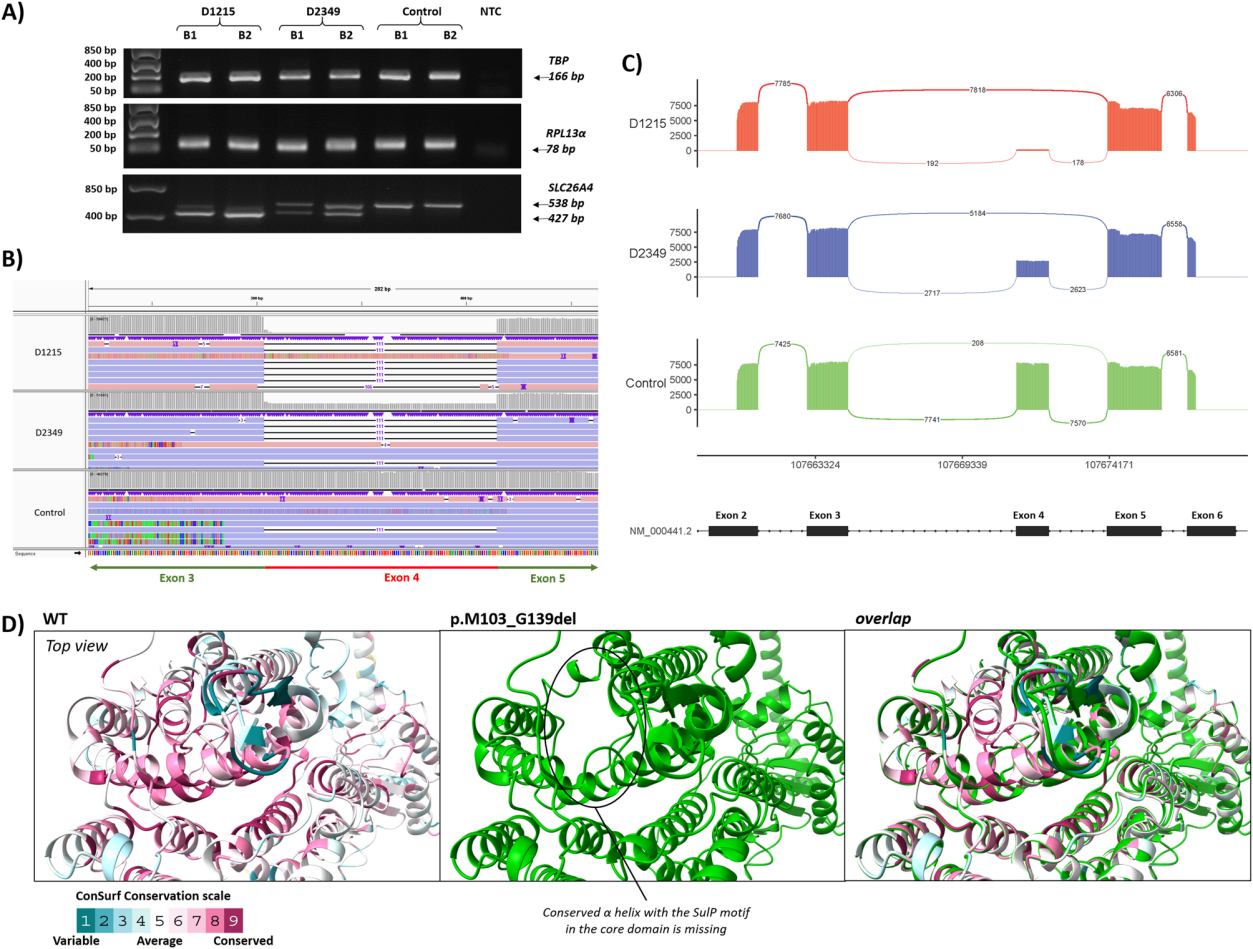
Effect of variant c.415G>A on pre-mRNA splicing in nasopharyngeal swab-derived samples.** A** Gel electrophoresis of PCR amplicons generated from cDNA transcribed from RNA of nasopharyngeal swabs. The samples were analysed in duplicates (B1 and B2). Results include endogenous *TBP* and *RPL13A* controls, along with amplicons spanning exons 2–6 of the *SLC26A4* gene. **B** IGV visualization of nanopore sequencing reads mapped to the *SLC26A4* reference transcript NM_000441.2, illustrating exon 4 skipping associated with variant c.415G>A. **C** Sashimi plot of reads aligned to the chr7:107,661,600–107,675,150 region of the reference genome, generated using *ggsashimi*. The x-axis shows the genomic region, while the y-axis represents the read count. Proband D1215 exhibits significant exon 4 skipping, while his father (D2349) shows intermediate coverage of exon 4. Sequencing of the control samples confirmed retention of exon 4 in the transcripts and minimal skipping rate. **D** Section of the top view of the SLC26A4 protein showing WT (left panel) with amino acids colored according to the ConSurf conservation score. The model with deletion of amino acids from M103 to G139 resulting from exon 4 skipping (middle panel) and its overlap with WT (right panel) show missing conserved α helix in the core domain

Our functional study’s pathogenicity assessment was based on the assumption that the putative missense variant c.415G>A alters the amino acid sequence (p.G139R). However, the c.415G>A substitution is located at the final nucleotide of exon 4. As this position is essential for spliceosome recognition (Jaganathan et al. 2019), we hypothesized it may affect pre-mRNA splicing. To explore this possibility, we performed the Nanopore sequencing due to its capacity to identify all potentially relevant alternative transcripts generated by the c.415G>A variant. Briefly, nasopharyngeal swabs were collected in duplicates from D1215 (compound heterozygote for variants c.1001+1G>A and c.415G>A), his father D2349 (heterozygote for c.415G>A) and a control sample. RNA was isolated, reverse transcribed, and the 538 bp region spanning exons 2–6 of the *SLC26A4* gene was amplified by PCR to see if the exon(s) are retained or skipped (spliced out). Gel electrophoresis of PCR amplicons revealed a truncated amplicon of the length of 427 bp in the samples D1215 and D2349 (Fig. 7A). In the subsequent step, all amplicons were sequenced, and sequencing data were aligned to the reference transcript NM_000441.2. Vizualization using IGV software revealed a 111 bp deletion representing exon 4 skipping in the majority of transcripts from proband D1215. In contrast, both D2349 and the control sample showed a mixture of transcript isoforms, including transcripts with intact exon 4 (Fig. 7B). To further assess splicing dynamics, nanopore reads were mapped to the human genome GRCh38 and alternative splicing within the region chr7:107,661,600–107,675,150 was visualized using a ggsashimi-generated sashimi plot. The analysis confirmed markedly elevated exon 4 skipping frequency in proband D1215, whereas exon 4 retention was observed in a substantial proportion of reads from D2349 and was dominant in the control sample (Fig. 7C). We found out that c.415G>A produced a truncated transcript that leads to the deletion of 37 amino acids (p.M103_G139del) in the highly conserved α helix within the core domain of pendrin (Fig. 7D).

### Classification of identified variants in the *SLC26A4* gene

Based on the results of functional tests and nanopore sequencing, we classified the identified variants into four groups according to the HL-EP specifications of the ACMG variant interpretation guidelines (Table 2).

**Table 2:** Classification of *SLC26A4* variants identified in this study

Nucleotide change	Amino acid change	ACMG classification criteria*	Final classification	DVD	The first report in the literature	Functional characterization
**c.140G>A** ^3^	**p.R47Q**	PM2_P, BS3_P	VUS	No	Novel	**This study**
**c.415G>A**	**p.G139R**	PM5_S, PM3, PP3, PP4, PS3_P, PM2_P	P^1^	No	Novel	**This study**
	**p.M103_G139del**	PVS1_S, PM3, PM2_P, PP4	LP^2^			
**c.441G>A**	**p.M147I**	PM5_S, PS3_P, PM2_P, PM3_P, PP3, PP4	LP	Yes	Jonard et al. 2010	**This study**
**c.481T>A**	**p.F161I**	PM3_S, PS3_P, PM2_P, PP4	LP	Yes	Batissoco et al. 2022	**This study**
c.707T>C	p.L236P	PM3_VS, PM5, PS3_P, PM2_P, PP3, PP4	P	Yes	Van Hauwe et al. 1998	Scott et al. 2000
c.890del	p.P297Qfs*6	PVS1, PM3_VS, PM2_P, PP4	P	Yes	Gillam et al. 2005	Gillam et al. 2005
c.1001+1G>A	p.?	PVS1, PM3_VS, PP1_S, PM2_P, PP4,	P	Yes	Coyle et al. 1998	N/A
c.1198del	p.C400Vfs*32	PVS1, PM3_VS, PP1_S, PM2_P, PP4	P	Yes	Everett et al. 1997	N/A
c.1226G>A	p.R409H	PM3_VS, PM5_S, PP1_M, PS3_P, PM2_P, PP3, PP4	P	Yes	Van Hauwe et al. 1998	Gillam et al. 2005
c.1246A>C	p.T416P	PM3_VS, PP1_M, PS3_P, PM2_P, PP3, PP4	P	Yes	Van Hauwe et al. 1998	Scott et al. 2000
c.1334T>G	p.L445W	PM3_VS, PP1_S, PS3_P, PM2_P, PP3, PP4	P	Yes	Van Hauwe et al. 1998	de Moraes et al. 2016
**c.1589A>C**	**p.Y530S**	PM3_S, PM5_S, PM2_P, PP3, PP4	P	Yes	Pryor et al. 2005	**This study**
c.1790T>C	p.L597S	PM3_S, PP3, PP4, BS1	VUS	Yes	Campbell et al. 2001	Choi et al. 2009b; Pera et al. 2008
c.2089+1G>A	p.?	PVS1, PM3_VS, PP1_M, PP4, PM2_P	P	Yes	Blons et al. 2004	N/A
**c.2260del**	p.D754Ifs*5	PVS1_S, PM2_P, PP4	LP	No	Novel	**This study**
c.2326C>T	p.R776C	PP4, BS1, BS3_P	LB	Yes	Pryor et al. 2005	de Moraes et al. 2016

The variants indicated in boldface were further characterized by functional studies

*DVD* Variant listed in Deafness Variation Database v.9 (https://deafnessvariationdatabase.org/); *P* pathogenic; *LP* likely pathogenic; *VUS* variant of unknown significance; *LB* likely benign; *_VS* very strong; *_S* strong; *_M* moderate; *_P* supporting; *N/A* not applicable

* - classified based on the ACMG criteria (Richards et al. 2015) with specifications from the ClinGen SVI Splicing Subgroup (Walker et al. 2023) and ClinGen Hearing Loss Variant Curation Expert Panel (2022), considering recommendations for interpreting the loss of function variants (Abou Tayoun et al. 2018) and specific criteria for hereditary hearing loss (Oza et al. 2018)

^1^classification before splicing analysis

^2^classification after splicing analysis

^3^variant not found in a patient with enlarged vestibular aqueduct

### Clinical manifestations

Among probands with an identified causal genotype (*n* = 13), seven manifested with PDS and six with NSEVA. All probands with PDS harboured biallelic *SLC26A4* variants (M2 genotype). Additionally, three probands with biallelic *SLC26A4* variants presented with NSEVA only. All three cases were younger than 15 years at the time of clinical evaluation; thus, later onset of thyroid disease phenotype cannot be excluded. Finally, the remaining three probands with a monoallelic *SLC26A4* variant (M1 genotype) and CEVA were also assessed as NSEVA cases based on normal thyroid morphology and function. When comparing the size and structure of the thyroid gland in probands with biallelic *SLC26A4* variants (M2) to a group of probands with a monoallelic variant combined with the CEVA haplotype or variant in the *KCNJ10* gene, we observed a significantly larger thyroid volume per m² of body surface area in the group with biallelic variants: 11.9 ± 6.8 vs. 3.3 ± 0.8 ml/m², *p* = 0.003 (Supplementary Table S5) and a more granular thyroid structure (Supplementary Fig. S1).

In most cases, the hearing loss was congenital or prelingual (11/13), progressive (9/13) and profound (10/13) at least in one ear (Table 1). Four subjects reported an association of hearing loss onset or sudden worsening of pre-existing hearing impairment with head trauma. All 13 probands had bilateral EVA detected by temporal bone imaging.

## Discussion

Identifying the genetic cause of NSEVA/PDS based on the presence of two pathogenic variants in the *SLC26A4* gene is often challenging, and the relative distribution of biallelic *SLC26A4* variants (M2 genotype), monoallelic variants (M1 genotype) and no detected *SLC26A4* variant (M0 genotype) varies across different populations. In North American Caucasian EVA patients, approximately 25% are M2, whereas in East Asia, 67–90% of EVA patients have M2 genotypes (Honda et al. 2022). The prevalence of M2 genotypes in Europe is similar to that in the American population. In Czechia, only 27.3% of EVA patients carried the M2 genotype, and 13.6% had the M1 genotype (Pourová et al. 2010). Similarly, Polish data reported M2 and M1 genotypes in 35% and 30% of EVA patients, respectively (Bałdyga et al. 2023). In contrast, an Austrian study found a much lower frequency of the M2 genotype, with only one patient harbouring pathogenic variants in the *SLC26A4* gene among 19 cases with EVA (Roesch et al. 2018). In our Slovak cohort of 37 probands with EVA, we confirmed the M2 genotype in 10 probands (27%), whereas the M1 genotype was identified in another nine probands (24%), while the patient with the likely benign variant was excluded from M1 genotype group. We identified no causal or VUS variant in the *SLC26A4* gene in 18 probands (49%).

A significant proportion of the genetic diagnostic gap observed in Caucasian EVA patients with the M1 genotype may be explained by the presence of the full-length CEVA haplotype. This haplotype was previously identified in 70% (7/10) of patients from a North American Caucasian population, as well as in all (6/6) Danish patients, in 86% (6/7) of monoallelic *SLC26A4* Polish patients and in 50% (8/16) of Dutch patients with monoallelic variants in *SLC26A4* (Chattaraj et al. 2017; Bałdyga et al. 2023; Smits et al. 2022). In our cohort, the full-length CEVA was detected in 33% of M1 probands (3/9). If we include only likely pathogenic and pathogenic variants in the M1 genotype, CEVA prevalence in our cohort increases to 42.9% (3/7). The frequency of the CEVA haplotype among normal-hearing Europeans was 3% (Chattaraj et al. 2017).

In total in the patients with EVA, we identified 15 distinct variants in the *SLC26A4* gene, including nine classified as pathogenic, four as likely pathogenic, one variants of uncertain significance, and one as likely benign (Table 2). Notably, the most common variant in the Slovak NSEVA/PDS cohort is the pathogenic splicing variant c.2089+1G>A, detected in three probands in the homozygous state, in one in a compound heterozygous state, in two in the *trans* position with CEVA haplotype and in one in a monoallelic state (Table 1). Interestingly, this variant is very rare in Central Europe (Banghova et al. 2008), highlighting potential regional differences in the distribution of specific *SLC26A4* variants, even among geographically proximate populations. The high prevalence of this variant could be explained by the founder effect, similar to what is observed with variants such as p.H723R and c.919–2A>G in East Asia (Tsukada et al. 2015). In addition to the previously documented pathogenic variants (p.L236P, c.1001+1G>A, p.C400Vfs*32, p.R409H, p.T416P, p.L445W) identified predominantly in European populations (Tsukada et al. 2015) our study revealed novel variants p.R47Q, c.415G>A, and p.D754Ifs*5 which were not yet reported (Table 2).

To investigate the effects and potential disease associations of previously uncharacterized and novel *SLC26A4* variants (p.R47Q, c.415G>A, p.M147I, p.F161I, p.Y530S and p.D754Ifs*5), we performed a functional characterization of these variants based on determination of iodide transport activity, total and plasma membrane pendrin expression level and subcellular localization of pendrin using confocal imaging. The functional assay assessing the I⁻/Cl⁻ transport capacity of pendrin also reliably reflects the HCO_3_⁻/Cl⁻ exchange activity (Dossena et al. 2006, 2011; Pera et al. 2008). Misfolding of pendrin variants and their retention in the ER leads to degradation via the ER-associated degradation (ERAD) pathway (Yoon et al. 2008; Jung et al. 2016) and defective plasma membrane targeting playing a key role in the pathogenesis of PDS (Rotman-Pikielny et al. 2002).

The VUS p.R47Q variant, identified in a patient with moderate hearing loss but without EVA, demonstrated no functional or localization abnormalities when compared to the wild-type pendrin. These findings suggest that p.R47Q is likely a rare non-causal variant (with MaxAF in the European non-Finnish population 0.00000087) without a direct role in the pathogenesis of hearing loss. Moreover, this variant is located in a non-conserved N-terminal region (Supplementary Fig. S2), supporting the probably benign character of this variant. However, reclassification of the variant would require the identification of more families for cosegregation analysis.

The p.G139R variant, predicted to result from nucleotide change c.415G>A identified in two unrelated probands with NSEVA, represents a novel, interesting variant that we have functionally characterized using various approaches. The previously identified amino acid substitutions p.G139V (Anwar et al. 2009) and p.G139A (Van Hauwe et al. 1998), affecting the same amino acid residue, were predicted to influence the stability of the anion-binding pocket, essential for the proper transport activity of the protein (Liu et al. 2023). A previous study has shown significantly impaired HCO_3_⁻/Cl⁻ and I⁻/Cl⁻ transport function for the p.G139A variant (Wasano et al. 2020). Since the non-polar glycine at position 139 of the protein is a highly conserved amino acid, a change to a positive charged arginine with a longer side chain could have a significant effect on the function of the protein (Supplementary Fig. S2). Indeed, the functional assay confirmed the significantly reduced transport activity of this variant compared to the wild-type protein (Fig. 3). Among all six analyzed pendrin variants, variant p.G139R exhibited the lowest transport efficiency, highlighting the critical role of this pendrin region in maintaining proper functionality. The markedly diminished transport capacity was associated with substantial retention in the ER, an almost complete absence of proper localization to the PM and dramatically reduced levels of pendrin both in the plasma membrane as well as at the whole-cell level. However, substitution c.415G>A is located at the last nucleotide of exon 4. Similarly, the already known and published variants p.G139V and p.G139A result from substituting the first nucleotide in exon 5 (c.416G>T and c.416G>C, respectively). Both positions affect pre-mRNA splicing patterns and could be considered splicing variants. Computational predicted values for the variant c.415G>A indicated the donor loss (SpliceAI score 0.85; MaxEnt_wt_ score 5.73 vs. MaxEnt_c.415G>A_ score − 0.87). To experimentally validate the in silico prediction, we analyzed the transcriptional profile directly in samples obtained from the nasopharyngeal swab of a proband D1215, who carries the c.415G>A variant in a compound heterozygous state along with the c.1001+1G>A variant, and his father D2349, a heterozygous carrier for the c.415G>A variant (Fig. 7). Nanopore sequencing confirmed in frame exon 4 skipping resulting in truncation of the protein by 37 amino acids (NP_000432.1:p.M103_G139del). This sequence extends into the terminal part of transmembrane domain (TMD) 1, the entire TMD2 and the beginning of TMD3. The non-canonical transmembrane domains formed by TMD3 and TMD10 are delimited by TMD2 and TMD9, where the conserved Y127 residue belonging to the SulP motif and E384 in the “Saier motif” interact with each other to stabilize the protein fold (Gorbunov et al. 2014; Bassot et al. 2017). The loss of SulP motif may destabilize the protein, cause the unfolding and retention of improperly folded pendrin mutants in the ER, alter its membrane insertion properties, and affect the ion transport properties of the mutated pendrin (Fig. 2 and Supplementary Fig. S2).

Previous in silico studies by Klarov et al. 2022 indicated that the p.M147I variant of the SLC26A4 protein, which is located in an evolutionarily conserved region (the α3-helix region of the TMD) (Fig. 2 and Supplementary Fig. S2), can lead to the manifestation of diseases associated with a functional disruption of the core domain of the pendrin protein. Our functional studies confirmed this prediction, as we observed a significantly reduced iodide anion transport capacity associated with significantly reduced levels of both total and PM pendrin expression levels. On the other hand, the substitution of methionine to isoleucine (both amino acids are neutral non-polar) may have a mild effect on a hydrophobic interaction of M147 with L494 at the region between the pendrin core and gate domain (Rapp et al. 2017), reflecting the partial retention of transport function observed in our functional study (Fig. 3).

Pendrin variant p.F161I has been previously identified in the L1 region of pendrin (Fig. 2 and Supplementary Fig. S2). This extracellular loop may play a significant role in defining the physiological functions of pendrin (Kuwabara et al. 2018). On the other hand, according to Grantham’s distance, the amino acids phenylalanine and isoleucine are very similar (*D* = 21), and substitution of phenylalanine might lead to less severe impairment of transport function while preserving the proper localization of pendrin in the plasma membrane. Although analysis of iodide anion transport efficiency confirmed a significant reduction in transport activity compared to wild-type pendrin, the ability of the variant to transport iodide was partially preserved compared to the control cells (Fig. 3). Co-localization experiments confirmed increased retention of the protein in the ER and decreased localization in the PM; however, these changes were the least pronounced among all the tested variants. The pathogenic nature of this variant may be primarily related to significantly reduced levels of total and PM pendrin expression.

Variant p.Y530S was previously identified in patients with both NSEVA and PDS (Pryor et al. 2005). It affects a residue conserved among the *SLC26A4* orthologs, indicating that this variant plays a role in the pathogenesis of NSEVA or PDS. Y530 forms a polar contact with H723 in maintaining the folding of sulphate transporter anti-sigma (STAS) domains STASβ2 and STASα5. There may also be a cation-π interaction between these two residues during the protein’s movement at the cytosolic side (Rapp et al. 2017). Mutation to serine would change the size of Y530, which could interfere with local folding (Supplementary Fig. S2). Choi et al. 2009b showed that this variant exhibits prominent ER retention. Our functional characterization confirmed these findings; the p.Y530S variant showed massive ER retention, the localization in the PM was significantly impaired and the total and PM expression was significantly reduced. Although the iodide transport function of this variant was decreased considerably compared to wild-type pendrin, it retained a partial transport function.

The frameshift variant c.2260del (p.D754Ifs*5) is located in the penultimate exon 20 and introduces a premature termination codon, resulting in a protein truncated by 3.46% (Fig. 2). This degree of truncation is insufficient to predict degradation via the nonsense-mediated mRNA decay (NMD) pathway, and this abnormal protein is likely to be retained in the cells (Abou Tayoun et al. 2018). The analyses of Rapp et al. 2017 suggested the presence of a cytosolic STAS domain encompassing amino acid residues 518–730 in the pendrin protein. Multiple mutations have been described in the STAS domain affecting the function of SLC26A4 (Rapp et al. 2017). However, less information is known about the C-terminal tail of the protein following the STAS domain. Our functional analysis demonstrated a significantly reduced iodide anion transport capacity compared to wild-type pendrin, confirming the crucial role of the C-terminal region in proper pendrin activity.

It is worth noticing that all the variants functionally characterized in this study (6/6) retained ion transport activity to some extent, and no loss-of-function variants were identified. Although some authors have shown that a minimal retained transport ability is sufficient to prevent thyroid dysfunction but not sensorineural deafness and EVA (Scott et al. 2000), our results have shown that variants with significantly retained transport ability can cause PDS. Namely, variant p.Y530S was identified in a compound heterozygous state with the pathogenic variant p.T416P in a proband with prelingual, progressive hearing loss, EVA and goiter. Similarly, we identified variant p.F161I in a compound heterozygous state with pathogenic variant c.1001+1G>A in proband D92 who had congenital profound hearing loss with EVA and goiter. In this case, however, a shortened CEVA was also identified. While the partial CEVA haplotype has already been used to explain NSEVA causality (Smits et al. 2022), we observed the presence of 9-SNPs CEVA in a homozygous state combined with the heterozygous likely pathogenic variant p.F161I in an asymptomatic subject (D1984, mother of D92) (Fig. 1A), challenging its direct association with NSEVA symptoms.

Recessive pathogenic variants in the *KCNJ10 * gene have been described in homozygous and compound heterozygous state in patients with a complex syndrome comprising seizures, sensorineural deafness, ataxia, impaired intellectual development and electrolyte imbalance (SESAME or EAST syndrome) (Scholl et al. 2009). Yang et al. 2009 identified mutations in the *KCNJ10* gene associated with deafness with enlarged vestibular aqueduct in probands from two families who also carried heterozygous mutations in the *SLC26A4* gene. The *KCNJ10* mutations reduced potassium conductance activity, which is critical for generating and maintaining the endocochlear potential (Yang et al. 2009). However, some studies have raised doubts regarding the involvement of *FOXI1* and *KCNJ10* in NSEVA (Landa et al. 2013; Zhao et al. 2014, 2019; Bernardinelli et al. 2025). In our study, we identified a variant of uncertain significance p.P101T in the *KCNJ10* gene along with a likely pathogenic variant p.D754Ifs*5 in the *SLC26A4* gene and a shortened CEVA haplotype, which involved only three SNPs in a proband with hearing impairment and EVA. In the proband’s mother, who was diagnosed with unilateral hearing loss along with ipsilateral cochlear hypoplasia, we identified variants in both the *SLC26A4* and *KCNJ10* genes; however, the CEVA haplotype was not identified. The role of the shortened CEVA haplotype thus remains unclear so far (Fig. 1B). The *KCNJ10*:p.P101T variant has not yet been reported in the literature in individuals affected with *KCNJ10*-related conditions; however, ClinVar contains an entry for this variant (Variation ID: 205827), and the Grpmax Filtering AF is 0.13% (gnomAD v4.1.0). The p.P101T variant is a non-conservative amino acid substitution, which is likely to impact secondary protein structure, as these residues differ in polarity and size. The available evidence is currently insufficient to determine the role of this variant in the disease. Therefore, it has been classified as a VUS according to the ACMG classification guidelines (BS1_P). Based on the cosegregation analysis, we cannot confirm a possible digenic cause of the disease, but further characterization of the variant in the *KCNJ10* gene will be needed to confirm its role in the disease pathogenesis.

Through a detailed WES analysis of M1 proband samples (D619, D900, D1827, D1966, D533, D1319, D1547, D1560, and D1888), as well as proband D1998 with an identified likely benign variant in the *SLC26A4* gene, we identified several VUS variants with autosomal dominant inheritance or compound heterozygous variants in genes with autosomal recessive inheritance (see Supplementary Table S4).

The most notable ones appear to be variants in *OPA1* and *MYO1A* genes with high REVEL score and low MAF and a previously reported variant in the *TJP2* gene. Variants in the *OPA1* gene are associated with autosomal dominant optic atrophy, but some *OPA1* mutations have additional clinical features, including hearing loss, ataxia, and peripheral neuropathy (Huang et al. 2009). In the sample of proband D533, we identified a rare variant c.821A>G with a REVEL score of 0.73, which could indicate the pathogenic potential of this variant. However, a co-segregation analysis of the variant within the family confirmed the presence of the variant also in the sample of the proband’s asymptomatic father. Moreover, no visual impairment occurs in the family; therefore, this variant was evaluated as non-causal.

Variants in the unconventional myosin gene, *MYO1A*, have been reported to cause autosomal dominant non-syndromic sensorineural hearing loss (Donaudy et al. 2003; Vona et al. 2014). Association of *MYO1A* variants with deafness was refuted as either the variants of *MYO1A* were identified in healthy controls or the deafness was explained by convincing variants of different genes (Eisenberger et al. 2014; Patton et al. 2016). However, the role of *MYO1A* in the development of hearing loss remains uncertain. Zhang et al. (2019), for instance, demonstrated the association of SNPs in the genes *CDH23*,* CX43*,* KCNMA1*,* MYO1A*,* MYO7A*, and *OTOG* with noise-induced hearing loss. In proband D1888, who carries a heterozygous variant in the *SLC26A4* gene, we detected the rare variant c.1436A>G in the *MYO1A* gene, which exhibits a high pathogenicity prediction based on in silico analysis. However, its role in the proband’s phenotype remains uncertain and requires further in-depth study.

The autosomal dominant variant in the *TJP2* gene (NM_004817.4:c.334G>A), identified in the proband D1319 in our study, is a known published variant (Kim et al. 2014). This variant segregated with autosomal dominant hearing loss in a Korean family. In silico modelling indicated destabilization of the protein structure and potential misfolding of the protein, emphasizing the pathogenic nature of this missense substitution. However, considering the high value of Grpmax Filtering AF (0.007019 in gnomAD v4.1.0, East Asia population), we believe it is a SNP that is particularly frequent in some Asian populations (Korea). Since another autosomal dominant missense variant in the gene *TJP2* was identified in a Caucasian female with sensorineural hearing loss and bilateral EVA recently (Bernardinelli et al. 2025), further studies need to be done to evaluate the pathogenicity of this variant.

Whole-exome sequencing (WES) analysis in the M1 probands revealed additional rare variants in 10 genes (Supplementary Table S4). Although all of these variants had REVEL scores below the 0.7 threshold, suggesting limited predicted pathogenicity according to Oza et al. 2018; they also exhibited low minor allele frequencies (MAF), making them candidates for further functional investigation. It is important to be aware of the limitations of the WES. These include the inability to detect variants in non-coding regions, such as deep intronic mutations, that may have regulatory functions or affect splicing. WES can also miss large structural variants and has reduced sensitivity in genomic regions with high GC content or low sequencing coverage. As a result, the presence of a second, undetected pathogenic variant in the *SLC26A4* gene - or in other genes associated with hearing loss - cannot be ruled out in M1 probands.

In our study we observed a clear relationship between the number of impaired alleles and symptoms severity. Most patients with biallelic pendrin variants (7/10) displayed an enlarged thyroid gland, three presented with NSEVA only, but were younger than 15 years (8–11 years) at the time of clinical evaluation; thus, later onset of the thyroid disease phenotype cannot be excluded. In contrast, all nine M1 probands exhibited only NSEVA symptoms. Chao et al. 2019 showed that the CEVA haplotype is associated with a less severe phenotype than alleles with a mutation affecting the coding regions or splice sites of *SLC26A4*. Despite the small number of patients with monoallelic mutations (M1) with CEVA in our study, the analysis confirmed significantly larger thyroid volume per m² of body surface area and more granular thyroid structure in the group with biallelic variants (M2) (Supplementary Fig. S1). Interestingly, among the 18 probands without identified causal *SLC26A4* variant, three exhibited PDS-like symptoms. The thyroid dysfunction observed in these probands may be independent of hearing impairment and EVA, suggesting the presence of a phenocopy of PDS, often referred to as Pseudo-Pendred syndrome, arising through distinct pathophysiological mechanisms (Fugazzola et al. 2002; Kara et al. 2010; Dossena et al. 2011). While hearing loss in PDS usually occurs congenitally or by early childhood and can progress over time, goiter can develop in adolescence or early adulthood.

On the other hand, we did not identify significant differences in the hearing loss phenotype, although this evaluation may be limited by the small number of patients with monoallelic mutations (M1) with CEVA, as mentioned above. These findings are in line with Smits et al. 2022 where no association of the CEVA allele with a milder HL compared to *SLC26A4* variants affecting the protein-coding sequences was found.

## Conclusion

In our study, a genetic cause of PDS and NSEVA was identified in 13 out of 37 probands (35%). Functional characterization not only confirmed the pathogenic potential of variants p.G139R, p.M147I, p.Y530S, p.D754Ifs* 5 and p.F161I but also extended the knowledge of molecular mechanisms of PDS and non-syndromic hearing loss. In addition, our results highlight the importance of characterizing potential splicing variants. Phenotypic characterization revealed that probands with biallelic *SLC26A4* pathogenic variants have significantly larger thyroid volume per m^2^ of body surface area than subjects with monoallelic *SLC26A4* variants and the CEVA haplotype.

## Supplementary Information

Supplementary Material 1.

## Data Availability

No datasets were generated or analysed during the current study.

## Abbreviations

ACMG: American College of Medical Genetics and Genomics
ANOVA: One-way analysis of variance
Anti-TG: Antithyroglobulin antibody
anti-TPO: Thyroid peroxidase antibody
BAM: Binary Alignment Map
BWA: Burrows-Wheeler Alignment tool
CEVA: Caucasian enlarged vestibular aqueduct-associated haplotype
ECFP: Enhanced cyan fluorescent protein
ER: Endoplasmic reticulum
ERAD: Endoplasmic reticulum-associated degradation
EVA: Enlarged vestibular aqueduct
EYFP: Enhanced yellow fluorescent protein
GATK: Genome Analysis Toolkit
HBSS: Hank’s balanced salt solution
HL: Hearing loss
HL-EP: Hearing Loss Variant Curation Expert Panel
IGV: Integrative Genomics Viewer
KASP: Kompetitive allele specific PCR
LB: Likely benign
LP: Likely pathogenic
NMD: Nonsense-mediated mRNA decay
NSEVA: Non-syndromic hearing loss with enlarged vestibular aqueduct
P: Pathogenic
PDS: Pendred syndrome
PM: Plasma membrane
SLC26A4: Solute Carrier Family 26 Member 4
SNP: Single-nucleotide polymorphism
STAS: Sulphate Transporter and AntiSigma factor antagonist
T3: Triiodothyronine
T4: Thyroxine
TMD: Transmembrane domain
TSH: Thyroid-stimulating hormone
VUS: Variant of unknown significance
WES: Whole exome sequencing
WT: Wild-type
