# Supplementary material for: Genetic heterogeneity in patients with enlarged vestibular aqueduct and Pendred syndrome
Source: Mol Med. 2025 May 27;31:208. doi: 10.1186/s10020-025-01262-x (PMC12107780; doi:10.1186/s10020-025-01262-x)
Supplement: Supplementary file 1 — Supplementary Material 1. [file 10020_2025_1262_MOESM1_ESM.docx]

**Genetic heterogeneity in patients with enlarged vestibular aqueduct and**

**Pendred syndrome.**

**Marek Sklenar^1^, Silvia Borecka^1^, Lukas Varga^1,2^, Emanuele Bernardinelli^3^, Juraj Stanik^1,4^, Martina Skopkova^1^, Miroslav Sabo^1^, Diana Ugorova^2^, Silvia Dossena^3,5^, Daniela Gasperikova^1*^**

^1^Diabgene Laboratory, Institute of Experimental Endocrinology, Biomedical Research Center, Slovak Academy of Sciences, Bratislava, Slovakia

^2^Department of Otorhinolaryngology - Head and Neck Surgery, Faculty of Medicine and University Hospital Bratislava, Comenius University, Bratislava, Slovakia

^3^Institute of Pharmacology and Toxicology, Paracelsus Medical University, 5020 Salzburg, Austria

^4^Department of Paediatrics, Faculty of Medicine and National Institute of Children’s Diseases, Bratislava, Slovakia

^5^Research and Innovation Center Regenerative Medicine & Novel Therapies, Paracelsus Medical University, 5020 Salzburg, Austria

**SUPPLEMENTARY INFORMATION**

**Supplementary methods**

***Thyroid function evaluation***

Thyroid gland size and structure were examined by neck ultrasonography and thyroid function was tested by a blood test for hormones, including TSH, T3 and T4, and anti-thyroglobulin (anti-TG) and anti-thyroid peroxidase (anti-TPO) antibodies. Body Surface Area corrected thyroid Volume (BSAV) was calculated based on total thyroid volume and the Du Bois method of BSA calculation (Du Bois et al. 1989).

$$BSAV (ml/m^{2})=\frac{thyroid volume}{0.007184 \mathrm{Height}^{0.725}\mathrm{Weight}^{0.425}}$$

***Genomic DNA isolation***

Patient whole blood was collected in the PAXgene Blood DNA Tubes (PreAnalytiX, Qiagen, Hilden, Germany) using a standard venipuncture technique. Total genomic DNA was isolated with the Whole Blood DNA Maxi Preparation Kit (Bioteke Corporation, Wuxi, China) or by an automatic isolator using the MagCore® Genomic DNA Large Volume Whole Blood Kit (RBC Bioscience, New Taipei City, Taiwan), according to the manufacturer’s instructions. The concentration of DNA was measured with a NanoDrop™ 2000/2000c spectrophotometer (Thermo Fisher Scientific, Waltham, MA, USA).

***Genomic DNA analysis***

Identification of variants in the SLC26A4 gene was performed at two tiers. First, we performed Sanger sequencing in all 37 samples. Next, we performed WES analysis in all individual without biallelic variants in the *SLC26A4* gene. Moreover, Sanger sequencing was performed to verify variants identified by WES, and to determine co-segregation of the candidate variants with hearing loss in all participating family members. All 21 exons and their flanking intronic regions were amplified by polymerase chain reaction (PCR) using the primers listed in Supplemental Table S1. Sequencing reactions of PCR products were carried out using BigDye Terminator v3.1 chemistry and separated on ABI 3500 genetic analyzer (Applied Biosystems, Thermo Fisher Scientific, Waltham, MA, USA) according to the manufacturer’s instructions. Sequence chromatograms were analyzed by the SeqScape Software v2.7 (Thermo Fisher Scientific). GenBank RefSeq NG_008489.1 and NM_000441.2 were used as the *SLC26A4* reference sequences.

Whole-exome sequencing (WES) of the genomic DNA was performed by the service providers (Theragen, Seongnam-si, Gyeonggi-do, Republic of Korea and Novogene, Cambridge, England, United Kingdom). Library preparation was conducted using the SureSelect XT V6 kit (Agilent Technologies, Santa Clara, CA, USA), and sequencing was carried out on either the HiSeq or NovaSeq Illumina platform (Illumina, San Diego, CA, USA).

The FASTQ files from the providers were processed using our in-house pipeline, employing the Genome Analysis Toolkit (GATK) to align reads to the GRCh37 reference genome with BWA (Li et al. 2009). We applied GATK (McKenna et al. 2010) for base quality score recalibration, indel realignment, duplicate removal, and conducted SNP and INDEL discovery and genotyping across all samples, using standard hard filtering parameters or variant quality score recalibration according to GATK Best Practices recommendations (DePristo et al. 2011; Van der Auwera et al. 2013).

Variants in the obtained VCF file (variant call format) were decomposed and normalized using software tool *vt normalize* (Tan et al. 2015) and subsequently annotated with respect to their potential effects on genes and transcripts with Variant effect predictor (McLaren et al. 2016) by adding scores from *in silico* prediction algorithms PolyPhen and SIFT (Adzhubei et al. 2010; Sim et al. 2012). Finally, the Gemini framework (Paila et al. 2013) was used for annotating variants with additional data from genome annotation databases. Common variants (with MAF ≥ 0.01) were filtered out and subsequent prioritization was performed manually based on annotation information. Identified variants were classified based on the American College of Medical Genetics and Genomics (ACMG) criteria (Richards et al. 2015) with specifications from the ClinGen SVI Splicing Subgroup (Walker et al. 2023) and ClinGen Hearing Loss Variant Curation Expert Panel (2022), considering recommendations for interpreting the loss of function variants (Abou Tayoun et al. 2018) and specific criteria for hereditary hearing loss (Oza et al. 2018). Candidate variants identified by WES and their co-segregation in all participating family members were verified by Sanger sequencing, as described above.

**RNA isolation and transcription**

RNA was isolated from the nasopharyngeal swabs of three objects using the QIAGEN RNeasy Mini Kit (Qiagen) protocol with DNaseI digestion step included to remove genomic DNA contamination. The RNA concentration was measured using a NanoDrop™ 2000/2000c spectrophotometer (Thermo Fisher Scientific). Subsequently, 1 µg of isolated RNA was reverse transcribed into cDNA using SuperScript III Reverse Transcriptase (Invitrogen, Thermo Fisher Scientific), according to the manufacturer′s instructions.

**Pendrin functional test**

The pendrin ion transport efficiency was monitored by measuring the iodide influx in cells expressing wild-type pendrin or pendrin variants by fluorometric analysis, as described previously (Dossena et al. 2006, Dror et al. 2010, Procino et al. 2013). Transfected HEK 293 Phoenix cells were initially washed and bathed in 70 μL of a high-chloride solution (in mM: KCl 2, NaCl 135, CaCl_2_ 1, MgCl_2_ 1, D-glucose 10, 4-(2-hydroxyethyl)-1-piperazineethane sulfonic acid (HEPES) 20, 308 mOsm/KgH_2_O adjusted with mannitol, pH 7.4), and the baseline fluorescence intensity was measured (1 measurement/sec for 3 sec). Subsequently, 140 μL of a high-iodide solution (in mM: KCl 2, NaI 135, CaCl_2_ 1, MgCl_2_ 1, D-glucose 10, HEPES 20, 308 mOsm/KgH_2_O adjusted with mannitol, pH 7.4) were injected into each well, and the fluorescence intensity was measured again (1 measurement/sec for 16 sec). The background fluorescence measured in cells transfected with 0.24 μg/well of the pTARGET vectors was subtracted from all of the other fluorescence measurements of the same 96-well plate. The endogenous iodide influx was determined in cells transfected with 0.12 µg/well of the empty pTARGET vector and 0.12 µg/well of the EYFP H148Q;I152L vector.

Following heterologous expression of wild-type pendrin or pendrin variants in cells, iodide enters the cytosol from the extracellular space, which will result in the quenching of iodide-sensitive EYFP H148Q;I152L fluorescence intensity. The transport efficiency of individual variants is therefore reflected by negative % fluorescence variations (ΔF%).

Fluorescence intensity was quantified with the VICTOR^TM^ X3 Multilabel Plate Reader (Perkin Elmer, Waltham, MA, USA) equipped with a liquid dispenser and the following filters: excitation: F485 (excitation center wavelength (CWL): 485 nm, bandwidth: 14 nm), emission: F535 (emission CWL: 535 nm, bandwidth: 25 nm).

**Determination of total expression level by confocal imaging**

Determination of total expression levels of pendrin variants was performed by quantitative confocal imaging as formerly described (de Moraes et al. 2016, Roesch et al. 2021, Matulevicius et al. 2022). Briefly, cells expressing SLC26A4-EYFP were fixed with 4% paraformaldehyde for 30 min, counterstained with 0.1 μg/mL 4′,6-diamidino-2-phenylindole (DAPI) for 10 min, subsequently washed and imaged in Hank’s balanced salt solution (HBSS, Sigma-Aldrich).

Imaging was performed with a Leica TCS SP5II AOBS confocal microscope (Leica Microsystems, Wetzlar, Germany) equipped with a HCX PL APO 63×/1.20 Lambda blue water immersion objective and controlled by the LAS AF SP5 software version 2.7.3.9723 (Leica Microsystems). EYFP was excited with the 514 nm line of the Argon laser, and emission was detected between 525 and 600 nm; DAPI was excited with a diode laser (405 nm), and emission was detected between 430 and 470 nm. Laser power and photomultipliers gain were kept rigorously constant for the acquisition of all images. The fluorescence intensity (in average levels of gray) of the whole imaging field in the EYFP emission window was subtracted for the background fluorescence and normalized for the background-subtracted fluorescence intensity in the DAPI emission window to determine pendrin expression levels normalized for the density of cells.

**Determination of** **expression level in the plasma membrane by confocal imaging**

The expression level of pendrin variants in the plasma membrane region was determined in living HeLa cells seeded in 6-well plates and co-transfected with 1.25 μg/well of a pEYFPN1 vector encoding for the wild-type or mutant fusion protein SLC26A4-EYFP and 0.25 μg/well of a pECFPC vector (Clontech) bearing the transfection marker enhanced cyan fluorescent protein (ECFP), as formerly described (de Moraes et al. 2016). To guide the choice of regions of interest (ROIs) of the plasma membrane, 72 h post-transfection cells were stained on ice with 1.25 μg/ml CellMask^TM^ Deep Red plasma membrane (C10046, Invitrogen Molecular Probes, Waltham, MA, USA) in HBSS for 5 min, washed, and imaged in ice-cold HBSS.

Imaging was performed with a Leica TCS SP5II AOBS confocal microscope as described above. EYFP was excited with the 514 nm line of the Argon laser, and emission was detected between 525 and 600 nm; ECFP was excited with a diode laser (405 nm) and emission was detected between 450 and 490 nm. CellMask^TM^ Deep Red stain was excited at 633 nm (HeNe laser) and emission was detected in the 643–750 nm range. Laser power and photomultipliers gain were kept rigorously constant for acquisition of all images.

To obtain wild-type or mutant pendrin expression levels in plasma membrane normalized for the transfection efficiency of the single cell, the intensity of EYFP fluorescence was measured in three plasma membrane ROIs, corrected for the background fluorescence and normalized for the background-subtracted ECFP fluorescence intensity measured in the cytosol of the same cell.

**Co-localization experiments**

The impact of pendrin variants on their subcellular localization was determined in living HeLa cells by co-localization of wild-type or mutant SLC26A4-EYFP and markers of the plasma membrane (CellMaskTM Deep Red plasma membrane stain, C10046, Invitrogen Molecular Probes) or endoplasmic reticulum (ER-TrackerTM Red, BODIPYTM TR glibenclamide, E34250, Invitrogen Molecular Probes), as formerly described (de Moraes et al. 2016, Matulevicius et al. 2022).

To stain the plasma membrane, cells were treated as described above. To stain the endoplasmic reticulum, cells were washed three times with Krebs-Henseleit buffer (Sigma-Aldrich) at room temperature, incubated for 20 min at 37 °C and 5% CO_2_ with 1 µM ER-Tracker^TM^ Red in Krebs-Henseleit buffer, washed again three times with Krebs-Henseleit buffer and immediately imaged in HBSS at room temperature.

Imaging was performed by sequential acquisition with a Leica TCS SP5II AOBS confocal microscope as described above. For co-localization with the plasma membrane, EYFP was excited with the 514 nm line of the Argon laser, and emission was detected in the 525–600 nm range; the CellMask^TM^ Deep Red stain was excited at 633 nm (HeNe laser) and emission was detected in the 643–750 nm range. For co-localization with the ER, EYFP was excited with the 514 nm line of the Argon laser and emission was detected in the 525–555 nm range; ER-Tracker^TM^ Red was excited at 561 nm (diode-pumped solid-state DPSS laser) and emission was detected in the 571–650 nm range.

Co-localization of pendrin variants with markers of plasma membrane or endoplasmic reticulum in single cells was quantified with the Colocalization tool of the LAS AF SP5 software (Leica Microsystems) and represented as the Pearson's correlation coefficient (Adler et al. 2010).

**Supplementary Table S1:** Primer sequences adopted from Coyle et al. 1998, Lofrano-Porto et al. 2008, Wu et al. 2010, used for the identification and verification of variants in the *SLC26A4* gene

| **Exon number** | **Primer sequence** | |
| --- | --- | --- |
|  | **Forward 5' - 3'** | **Reverse 5' - 3'** |
| 1 | TTC CTC TTC TCC TCC CCA TG | GTG TGG GCA TCT TCA GGG |
| 2 | GGC TGC AGC TAA CAG GTG ATC | GAG GAC CGG AGA CCG AAA GTC |
| 3 | ACA GTT CTT GGC AAA AGC ATG G | GAA GGG TAA GCA ACC ATC TGT CAC |
| 4 | TAA TCA CTT TGC ATG TGC TTT | GCC AAA ACA CTT TAA ACA TGA GCA |
| 5 | CTC AGC TTC TTT CGT GAA CAA AC | TTT GGG TTC CAG GAA ATT ACT TTG T |
| 6 | GTG CTA TAG GCA GGC TAC TAG TGT T | CCT GGC CCA GAC TCA GAG AAT |
| 7 | TGG GAA GAT TCA TAT GAG AAT TGA TTG | TGG TTG TTT CTT CCA GAT CAC A |
| 8 |  |  |
| 9 | CAT GTG AAA TGG CAT GGA TGG | GGT CTG GTG AAA GAA TCC AAC C |
| 10 | CGC AGA GTA GGC ATG GGA GTT T | TTG TCC TGC TAA GCT CGG TGC |
| 11 | AGA CAG GGA AGT ATG AAG TGT G | TTT CTC CTC TGG AGT TCC CAA A |
| 12 |  |  |
| 13 | AGG TAG TTA TCA CAT GAT GGT ACC TG | GAG CAC AGC AGT AGA GGA CAT |
| 14 | AAA CAA CAG AAT GAT GGG CTC | GTC AGA AGG TGC ACT GGA TC |
| 15 | CCC AGA CAA TTT CTT TTA ATG C | TTG GAC CCC AGT AAA TAC TTG T |
| 16 | CCT TTG AGA AAT AGC CTT TCC AG | GCT CTC ATC AGG GAA AGG AA |
| 17 | AGT TTG GGC TGA GGT GAA ACC | CAA AGC CCA TGT ATT TGC CCT G |
| 18 | TCC TGA GCA AGT AAC TGA ATG C | GAA AGG GCT TAC GGG AAA GT |
| 19 | TTT CTT AGC TGG GCA TGG TAG G | GGA ATT TAT GTA CAC AAA TCC CAG ATC AC |
| 20 | AGA AGC ACC AGG AAA GCT TCA | GGG AAT TAT GTT CCC TGA CAG TTC |
| 21 | CCT AAG ATG AGT AGC AGT AAG CA | GCT GCC AAA TCG TCT GAA TAA TTC |

**Supplementary Table S2:** Whole exome sequencing dataset (virtual panel) of 579 genes associated with hearing loss

*A1BG*

*ABCC1*

*ABHD12*

*ABHD5*

*ACOX1*

*ACSL4*

*ACTB*

*ACTG1*

*ADCY1*

*ADGRL4*

*ADGRV1*

*AFG2A*

*AGAP1*

*AIFM1*

*AK2*

*ALG12*

*ALMS1*

*AMMECR1*

*ANKH*

*ANKRD24*

*ANLN*

*AP1S1*

*AP3B1*

*APPL1*

*AQP5*

*ARID1B*

*ARSG*

*ASAH1*

*ASPA*

*ATOH1*

*ATP11A*

*ATP12A*

*ATP1A2*

*ATP1A3*

*ATP2B2*

*ATP2B4*

*ATP6V0A4*

*ATP6V1B1*

*ATP6V1B2*

*ATP8B1*

*BARHL1*

*BAZ1B*

*BBS10*

*BBS9*

*BCAP31*

*BCS1L*

*BDNF*

*BDP1*

*BMP4*

*BRD2*

*BSN*

*BSND*

*BTD*

*BTK*

*CABP2*

*CACNA1D*

*CALB1*

*CALB2*

*CALM1*

*CAMK2B*

*CAMSAP3*

*CATSPER2*

*CCDC50*

*CCNQ*

*CD151*

*CD164*

*CDC14A*

*CDC42*

*CDH19*

*CDH23*

*CDIP1*

*CDK14*

*CDK5RAP2*

*CDK9*

*CDKN1C*

*CEACAM16*

*CELF2*

*CEP250*

*CEP78*

*CGN*

*CHD4*

*CHD7*

*CHM*

*CHN1*

*CHST15*

*CHSY1*

*CIB2*

*CISD2*

*CKMT1B*

*CLCNKA*

*CLCNKB*

*CLDN10*

*CLDN14*

*CLDN9*

*CLIC2*

*CLIC5*

*CLIC6*

*CLPP*

*CLPX*

*CLRN1*

*CNTN4*

*COA8*

*COCH*

*COL11A1*

*COL11A2*

*COL1A1*

*COL1A2*

*COL2A1*

*COL4A3*

*COL4A4*

*COL4A5*

*COL4A6*

*COL9A1*

*COL9A2*

*COL9A3*

*COQ2*

*COQ6*

*COX10*

*CRYL1*

*CRYM*

*CSNK1G3*

*CTBP2*

*CTTN*

*CXCR2*

*DCAF17*

*DCDC2*

*DCHS1*

*DCHS1*

*DENND2B*

*DIABLO*

*DIAPH1*

*DIAPH3*

*DLX5*

*DLX6*

*DMXL2*

*DNAH8*

*DNAJC3*

*DNM1L*

*DNMT1*

*DSPP*

*DUOXA2*

*EDN3*

*EDNRA*

*EDNRB*

*EFTUD2*

*EHMT1*

*EIF3F*

*ELMOD3*

*EMG1*

*ENTREP1*

*EPS8*

*EPS8L1*

*EPS8L2*

*ERAL1*

*ERCC2*

*ERCC3*

*ERCC5*

*ESPN*

*ESRP1*

*ESRRB*

*EYA1*

*EYA2*

*EYA4*

*FADS3*

*FAM107B*

*FAM136A*

*FAM20C*

*FARS2*

*FBXO33*

*FCHSD1*

*FDXR*

*FERRY3*

*FGF10*

*FGF13*

*FGF3*

*FGFR1*

*FGFR2*

*FGFR3*

*FITM2*

*FLNB*

*FLRT3*

*FOXC1*

*FOXF2*

*FOXI1*

*FREM2*

*FZD10*

*FZD9*

*GAB1*

*GAS2*

*GATA2*

*GATA3*

*GDF6*

*GDNF*

*GFI1*

*GGPS1*

*GIPC3*

*GJA1*

*GJB1*

*GJB2*

*GJB3*

*GJB4*

*GJB5*

*GJB6*

*GJC3*

*GLA*

*GNAI3*

*GPR152*

*GPSM2*

*GRAP*

*GREB1L*

*GRHL2*

*GRIP1*

*GRXCR1*

*GRXCR2*

*GSDME*

*GSK3B*

*GSTP1*

*GTPBP3*

*GYG1*

*GYPC*

*GZMK*

*HAAO*

*HARS1*

*HARS2*

*HDAC8*

*HES5*

*HGF*

*HMX1*

*HMX2*

*HOMER2*

*HOXA1*

*HOXA2*

*HOXB1*

*HS6ST1*

*HSD17B4*

*HYLS1*

*IARS1*

*IARS2*

*IDUA*

*IFNLR1*

*IGBP1*

*IGF1*

*IGFALS*

*IL17RD*

*IL6*

*ILDR1*

*JAG1*

*KARS1*

*KCNE1*

*KCNE5*

*KCNG1*

*KCNJ1*

*KCNJ10*

*KCNJ16*

*KCNQ1*

*KCNQ4*

*KDM6A*

*KIT*

*KITLG*

*KLC2*

*KLHL18*

*KMT2D*

*KPTN*

*LAMA2*

*LAMA5*

*LARS2*

*LGALS12*

*LGI2*

*LHFPL5*

*LHX3*

*LMO7*

*LMX1A*

*LORICRIN*

*LOXHD1*

*LOXL3*

*LRIG1*

*LRP2*

*LRP5*

*LRRTM1*

*LRTOMT*

*MAN2B1*

*MANBA*

*MAP2K1*

*MAP2K2*

*MARS2*

*MARVELD2*

*MASP1*

*MCM2*

*MCPH1*

*MED12*

*MET*

*MFN2*

*MGP*

*MINAR2*

*MIR182*

*MIR183*

*MIR96*

*MITF*

*MKRN2*

*MN1*

*MPO*

*MPZ*

*MPZL2*

*MRPS12*

*MSRB3*

*MT-ATP6*

*MT-ATP8*

*MT-CO1*

*MT-CO2*

*MT-CO3*

*MT-CYB*

*MTHFR*

*MT-ND1*

*MT-ND2*

*MT-ND3*

*MT-ND4*

*MT-ND4L*

*MT-ND5*

*MT-ND6*

*MTO1*

*MT-RNR1*

*MT-RNR2*

*MT-TA*

*MT-TC*

*MT-TD*

*MT-TE*

*MT-TF*

*MT-TG*

*MT-TH*

*MT-TI*

*MT-TK*

*MT-TL1*

*MT-TL2*

*MT-TM*

*MT-TN*

*MT-TP*

*MT-TQ*

*MT-TR*

*MT-TS1*

*MT-TS2*

*MT-TT*

*MT-TV*

*MT-TW*

*MT-TY*

*MUC5B*

*MYH14*

*MYH15*

*MYH2*

*MYH7B*

*MYH9*

*MYO15A*

*MYO1A*

*MYO1C*

*MYO1E*

*MYO1F*

*MYO3A*

*MYO5B*

*MYO6*

*MYO7A*

*MYO9A*

*MYOF*

*NARS1*

*NARS2*

*NCOA2*

*NDP*

*NDRG1*

*NEFL*

*NEUROG1*

*NF2*

*NLRP3*

*NOG*

*NOTCH1*

*NOTCH2*

*NR1D1*

*NR2F1*

*NTF3*

*NXF1*

*OCM*

*OFD1*

*OGDHL*

*OPA1*

*OPA3*

*OSBPL2*

*OTOA*

*OTOF*

*OTOG*

*OTOGL*

*OTX1*

*P2RX2*

*PAX1*

*PAX2*

*PAX3*

*PAX6*

*PAX9*

*PBX1*

*PCARE*

*PCDH15*

*PCGF2*

*PDE1C*

*PDE6B*

*PDSS1*

*PDZD7*

*PET100*

*PEX1*

*PEX11B*

*PEX12*

*PEX13*

*PEX14*

*PEX19*

*PEX2*

*PEX26*

*PEX3*

*PEX5*

*PEX6*

*PEX7*

*PHF20*

*PHYH*

*PI4KB*

*PISD*

*PJVK*

*PLCB4*

*PLS1*

*PMP22*

*PNPT1*

*POLD1*

*POLD3*

*POLG*

*POLR1C*

*POLR1D*

*POU3F4*

*POU4F3*

*PPIP5K2*

*PRDM5*

*PRKAR1A*

*PROK2*

*PROKR2*

*PRPS1*

*PRPS2*

*PSIP1*

*PSMC3*

*PTPN11*

*PTPRQ*

*PXMP2*

*QSOX2*

*RAB40AL*

*RAF1*

*RAI1*

*RBM10*

*RDX*

*REST*

*RET*

*RIPOR2*

*RMND1*

*RNASE12*

*RNASE3*

*RNASEH2B*

*RNF220*

*ROR1*

*RPS6KA3*

*RRM2B*

*S1PR2*

*SALL1*

*SALL4*

*SCD5*

*SELENOK*

*SEM1*

*SEMA3E*

*SERAC1*

*SERPINB6*

*SERPINF1*

*SETD5*

*SF3B4*

*SFI1*

*SGPL1*

*SH3TC2*

*SIX1*

*SIX2*

*SIX5*

*SLC12A1*

*SLC12A2*

*SLC17A7*

*SLC17A8*

*SLC19A2*

*SLC22A4*

*SLC25A21*

*SLC26A4*

*SLC26A5*

*SLC29A3*

*SLC33A1*

*SLC4A11*

*SLC52A2*

*SLC52A3*

*SLC5A5*

*SLITRK6*

*SMAD4*

*SMPX*

*SNAI2*

*SNX10*

*SOS1*

*SOX10*

*SOX2*

*SPATC1L*

*SPNS2*

*SRSF7*

*STAG2*

*STRC*

*SUCLA2*

*SUCLG1*

*SUN1*

*SYNE4*

*TAOK1*

*TBC1D24*

*TBL1X*

*TBL1Y*

*TBX1*

*TBX2*

*TBX22*

*TBX4*

*TCF21*

*TCIRG1*

*TCOF1*

*TECTA*

*TECTB*

*TFAP2A*

*TFB1M*

*TGFB1*

*TIMM8A*

*TIMM8B*

*TJP2*

*TK2*

*TLR10*

*TMC1*

*TMC2*

*TMEM126A*

*TMEM132E*

*TMEM164*

*TMIE*

*TMPRSS3*

*TMPRSS4*

*TMPRSS5*

*TMTC2*

*TNC*

*TNFRSF11B*

*TNFSF11*

*TPCN2*

*TPO*

*TPRN*

*TRAM2*

*TRIOBP*

*TRMU*

*TRPM2*

*TRPV4*

*TRRAP*

*TSFM*

*TSHZ1*

*TSPEAR*

*TUBB4B*

*TWIST1*

*TWNK*

*TYR*

*UBE3B*

*UBR1*

*USH1C*

*USH1G*

*USH2A*

*USP42*

*USP48*

*VPS13B*

*VPS33B*

*WBP2*

*WFS1*

*WHRN*

*XYLT2*

*YAP1*

*YWHAE*

*ZAN*

*ZCCHC14*

*ZNF469*

*ZNF664*

**Supplementary Table S3:** Sequences of mutagenesis primers

| ***SLC26A4* variant** | **Primer sequence** | |
| --- | --- | --- |
|  | **Forward 5' - 3'** | **Reverse 5' - 3'** |
| ***c.140G>A* (p.R47Q)** | gcc agg ctc tcc tgc agc gtc ttg c | gca aga cgc tgc agg aga gcc tgg c |
| ***c.415G>A* (p.G139R)** | cac tgg aaa agg tct aac tga gat atg tct tga tgt tcc aa | ttg gaa cat caa gac ata tct cag tta gac ctt ttc cag tg |
| ***c.441G>A* (p.M147I)** | caa cag atc cca cta tta aac tca cca ctg gaa aag gt | acc ttt tcc agt ggt gag ttt aat agt ggg atc tgt tg |
| ***c.481T>A*** **(p.F161I)** | gct gga tac gag aat gtg ttc gtc ggg gg | ccc ccg acg aac aca ttc tcg tat cca gc |
| ***c.1589A>C* (p.Y530S)** | ttg taa ttc ttg gta ctt ttg gag ata tct gtg cta ggg atg c | gca tcc cta gca cag ata tct cca aaa gta cca aga att aca a |
| ***c.2260del* (p.F754Ifs*5)** | tat taa ttc aag ggt att tta caa tcc tga atg aga gtg atc gtt tct | aga aac gat cac tct cat tca gga ttg taa aat acc ctt gaa tta ata |

**Supplementary results**

**Supplementary Table S4:** Candidate genes identified in M1 probands using whole exome sequencing

| **Patient**  **ID** | **Gene** | **Transcript** | **Chr position  (Assembly: GrCh38)** | **Genotype** | **cDNA** | **protein** | **RS ID** | **Allele  frequency** | **REVEL** | **SpliceAI** | **Inheritance** | **Classification** |
| --- | --- | --- | --- | --- | --- | --- | --- | --- | --- | --- | --- | --- |
| **D619** | ***COL1A1*** | NM_000088.4 | Chr17:50196165 | Het | c.992C>T | p.A331V | rs762653813 | 0.00001533 | 0.59 | 0.02 | AD | VUS |
|  | ***IFNLR1*** | NM_170743.4 | Chr1:24159608 | Het | c.532_535dup | p.G179Afs*37 | rs772082692 | 0.00001107 | N/A | 0.01 | AD | VUS |
|  | ***KCNJ10*** | NM_002241.5 | Chr1:160042232 | Het | c.301C>A | p.P101T | rs375361490 | 0.001279 | 0.407 | 0 | AR, DR | VUS |
|  | ***MYO1C*** | NM_001080779.2 | Chr17:1482899 | Het | c.508G>A | p.V170M | rs146251319 | 0.00006767 | 0.662 | 0 | AD | VUS |
| **D1827** | ***P2RX2*** | NM_170682.4 | Chr12:132618985 | Het | c.169G>A | p.V57M | N/A | 0 | 0.167 | 0.25 | AD | VUS |
| **D533** | ***OPA1*** | NM_130837.3 | Chr3:193631643 | Het | c.821A>G | p.Q274R | rs1258694003 | 2.800e-7 | 0.73 | 0.01 | AD | VUS |
|  | ***TSHZ1*** | NM_001308210.2 | Chr18:75285922 | Het | c.515C>T | p.T172M | rs961482761 | 0.00005454 | 0.078 | 0 | AD | VUS |
| **D1319** | ***MITF*** | NM_001354604.2 | Chr3:69964957 | Het | c.1290C>G | p.N430K | rs982952310 | 0.00001063 | 0.147 | 0.01 | AD, AR | VUS |
|  | ***TJP2*** | NM_004817.4 | Chr9:69218351 | Het | c.334G>A | p.A112T | rs144396411 | 0.007019 | 0.776 | 0 | AD | VUS |
| **D1547** | ***OPA3*** | NM_025136.4 | Chr19:45584650 | Het | c.115A>G | p.T39A | rs942993720 | 2.800e-7 | 0.374 | 0 | AD | VUS |
| **D1560** | ***SERPINF1*** | NM_002615.7 | Chr17:1771873 | Het | c.441G>C | p.K147N | rs138341386 | 0.0003171 | 0.036 | 0 | AD | VUS |
| **D1888** | ***FGFR1*** | NM_023110.3 | Chr8:38426125 | Het | c.742G>T | p.V248L | N/A | NF | 0.289 | 0.01 | AD | VUS |
|  | ***MYO1A*** | NM_005379.4 | Chr12:57038906 | Het | c.1436A>G | p.H479R | rs200230941 | 0.00001030 | 0.823 | 0.02 | AD | VUS |
|  | ***S1PR2*** | NM_004230.4 | Chr19:10224311 | Het | c.595T>C | p.S199P | rs1599235147 | NF | 0.518 | 0 | AR | VUS |
|  |  |  | Chr19:10224341 | Het | c.565C>T | p.H189Y | rs150504911 | NF | 0.141 | 0 | AR | VUS |

The allele frequency according to the GnomAD v4.1.0 database; the REVEL score ≥ 0.7 represents supportive evidence of pathogenicity, REVEL score ≤ 0.15 represents supportive evidence of benign; SpliceAI scores reflect the probability that the variant affects splicing at any position within a window around it (+/- 50bp). The value ≥0.2 indicates a low-confidence prediction, ≥ 0.5 suggests a moderate impact on splicing and ≥ 0.8 represents a high-confidence prediction of significant splicing alteration (Jaganathan et al. 2019); classification based on the ACMG criteria (Richards et al. 2015) with specifications from the ClinGen SVI Splicing Subgroup (Walker et al. 2023) and ClinGen Hearing Loss Variant Curation Expert Panel (2022), considering recommendations for interpreting the loss of function variants (Abou Tayoun et al. 2018) and specific criteria for hereditary hearing loss (Oza et al. 2018); Het – heterozygote, NF – not found, AD – autosomal dominant, AR – autosomal recessive, DR – digenic inheritance, VUS – variant of uncertain significance, N/A - not available.

**Supplementary Table S5:** Clinical and laboratory data of thyroid examination of probands with identified variants in the *SLC26A4* gene

|  | **Proband ID** | **Age at examination (years)** | **LLV of thyroid (ml)** | **RLV of thyroid (ml)** | **Total thyroid volume (ml)** | **Goiter** | **TSH ref. range 0.51-4.30 (mlU/l)** | **fT4 ref. range 10.40-21.36 (pmol/l)** | **T3 ref. range 3.88-8.02 (pmol/l)** | **anti-TG ref. range 0.0-64.0 (kIU/l)** | **anti-TPO ref. range 0.0-26.0 (kIU/l)** | **Therapy with  l-thyroxin**  **(µg/day)** | **Other clinical symptoms and comorbidities** | **Height (cm)** | **Weight  (kg)** | **BSAV**  **(ml/m^2^)** |
| --- | --- | --- | --- | --- | --- | --- | --- | --- | --- | --- | --- | --- | --- | --- | --- | --- |
| **Biallelic *SLC26A4* cases (M2)** | | | |  |  |  |  |  |  |  |  |  |  |  |  |  |
|  | D92 | 30 | N/A | N/A | 16.27 | Yes | 1.35 | 12.5 | N/A | N/A | N/A | No |  | 168 | 55 | 10.04 |
|  | D253 | 36 | 7.31 | 11.62 | 18.93 | Yes | 1.7 | 17.5 | N/A | N/A | N/A | 37.5 |  | 183 | 65 | 10.23 |
|  | D534 | 36 | 10.2 | 17.6 | 27.8 | Yes | 0.738 | 17.28 | 5.40 | <0.29 | 0.54 | No |  | 175.7 | 77 | 14.40 |
|  | D632 | 14 | 11.7 | 8.7 | 20.4 | Yes | 1.28 | 12.74 | 6.20 | 18.6 | 12.0 | No |  | 163.6 | 68 | 11.72 |
|  | D642 | 12 | 7.9 | 8.2 | 16.1 | Yes | 0.70 | 12.22 | 6.91 | 18.7 | 15.5 | No |  | 174 | 50.7 | 10.00 |
|  | D743 | 14 | 27 | 36 | 63 | Yes | 0.81 | 21.38 | 5.45 | 11.0 | 13.0 | 75 |  | 190.2 | 90.5 | 28.77 |
|  | D930 | 11 | 3.8 | 4.8 | 8.6 | No | 4.8 | 15.5 | 6.48 | 20 | 10.9 | No |  | 150 | 57.4 | 5.70 |
|  | D1215 | 8 | 5.1 | 5.6 | 10.7 | No | 1.96 | 13.45 | 6.62 | 17.0 | 8.0 | No |  | 135.6 | 41.6 | 8.63 |
|  | D1450 | 48 | N/A | N/A | N/A | Yes | N/A | N/A | N/A | N/A | N/A | 175 |  | 173 | 108 | N/A |
|  | D2128 | 10 | 5.4 | 8.3 | 13.7 | No | 1.75 | 18.28 | 6.52 | 21.0 | 12.3 | No |  | 167.5 | 68.2 | 7.74 |
| **Monoallelic *SLC26A4* cases (M1) with CEVA haplotype** | | | | | | |  |  |  |  |  |  |  |  |  |  |
|  | D900 | 12 | 2.0 | 4.6 | 6.6 | No | 1.7 | 11.6 | N/A | 15.5 | 15.9 | No | Type 1 Diabetes | 167.6 | 84 | 3.40 |
|  | D1827 | 7 | 1.7 | 1.4 | 3.1 | No | 1.86 | 14.82 | 6.87 | 20.2 | 17.7 | No |  | 135.3 | 27.5 | 2.98 |
|  | D1966 | 10 | 1.5 | 1.6 | 3.1 | No | 2.4 | 16.1 | N/A | N/A | N/A | No |  | 144 | 40.8 | 2.42 |
| **Monoallelic *SLC26A4* case (M1) with partial CEVA haplotype and *KCNJ10* variant** | | | | | | | | | | |  |  |  |  |  |  |
|  | D619 | 18 | 3.3 | 4.1 | 7.4 | No | 2.87 | 14.41 | 4.69 | 20.2 | 18.7 | No |  | 165.6 | 60.8 | 4.40 |

LLV – left lobe volume, RLV – right lobe volume, BSAV - Body surface area corrected thyroid volume, N/A – not available

**Supplementary Figure S1**


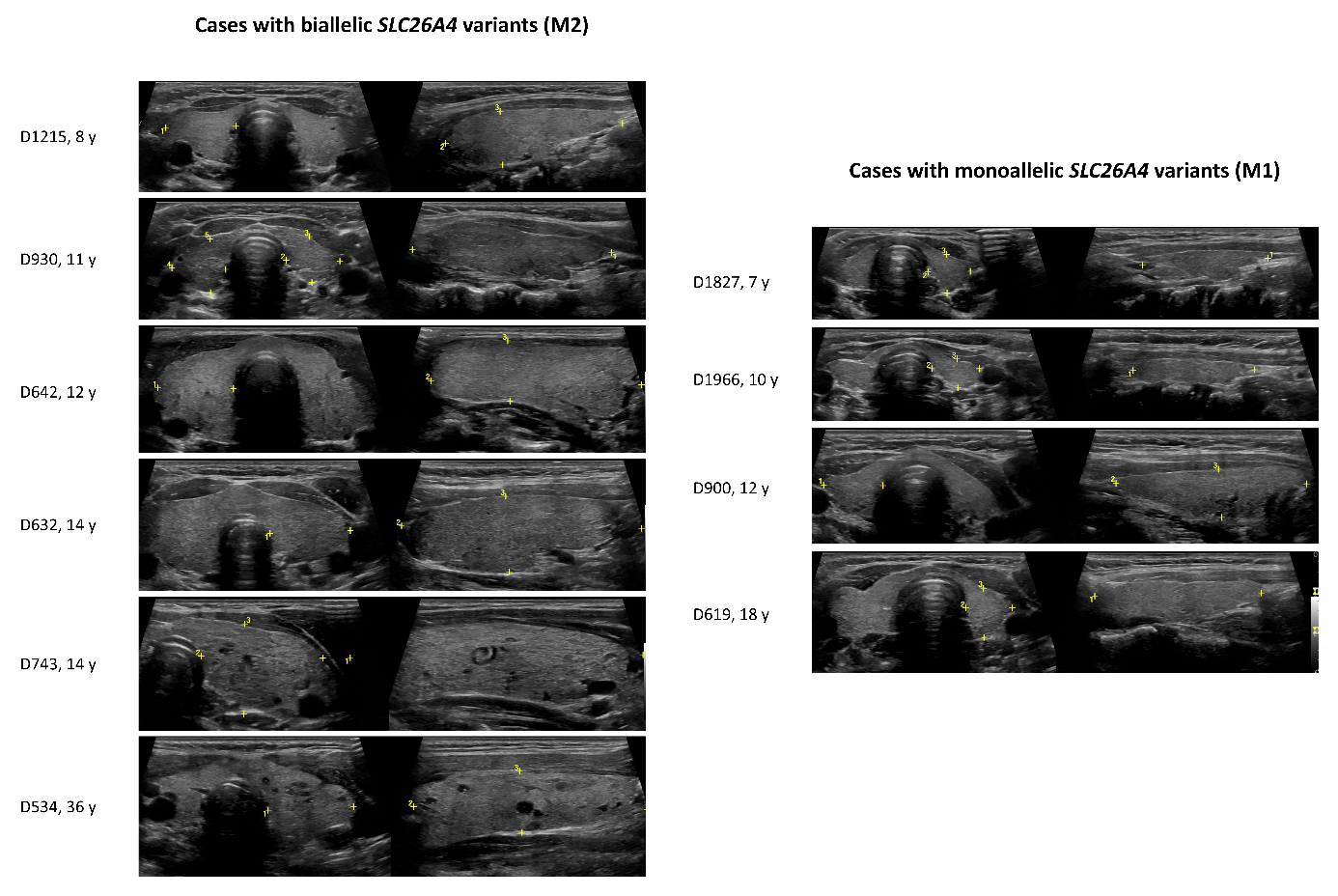


**Supplementary Fig. S1: USG of the thyroid gland of individuals from the cohort.** In the left column are individuals with biallelic variants in the SLC26A4 gene (M2), in the right column are individuals with monoallelic variants in the SLC26A4 gene (M1) and complete CEVA haplotype (D1827, D1966, D900) or variant in the gene KCNJ10 (D619). Individuals are arranged by age at examination in ascending order. In M2 patients, a larger thyroid volume is evident at each age as well as a more granular thyroid structure with accentuated follicular pattern (follicular goiter).

**Supplementary Figure S2**


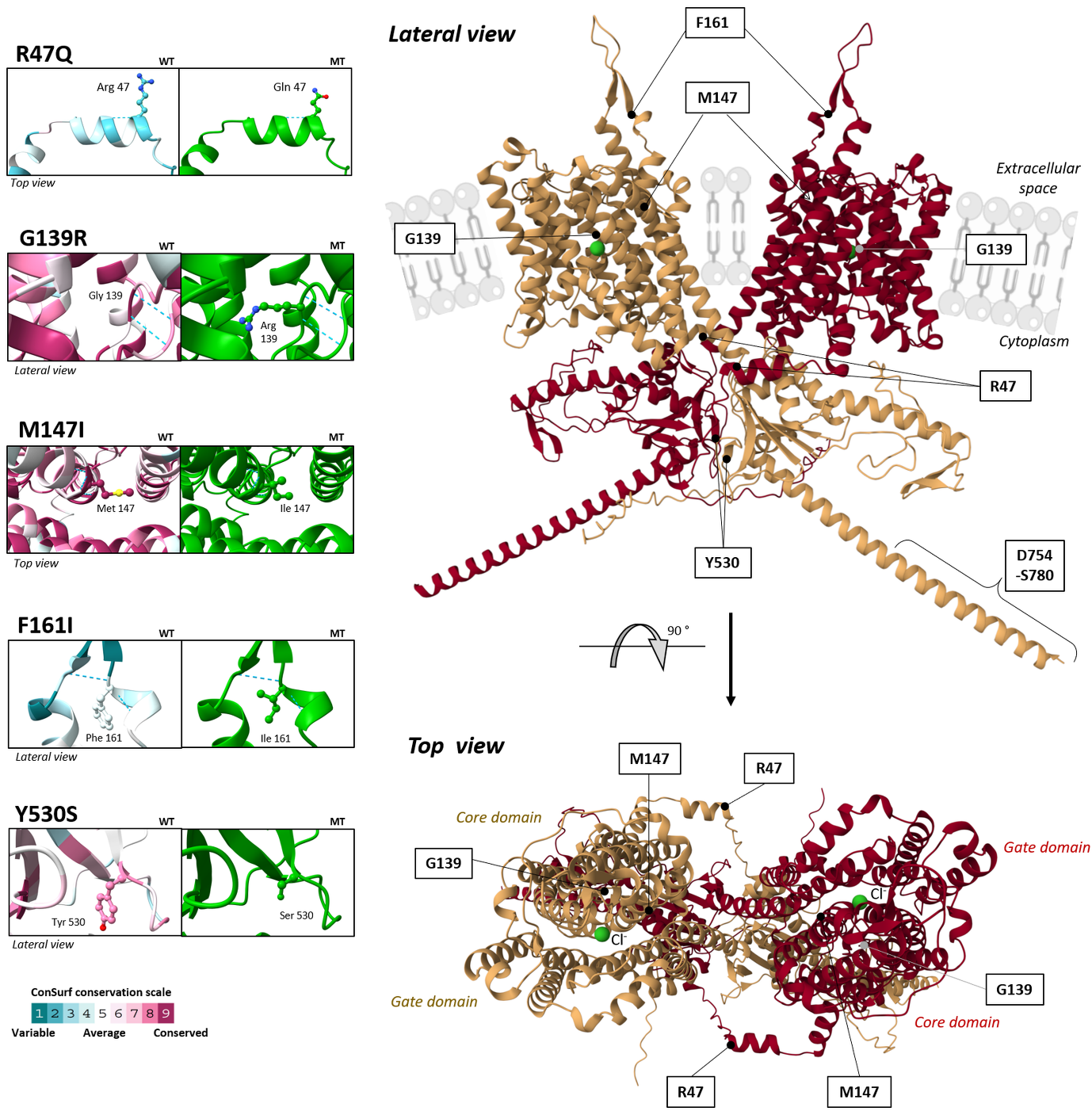


**Supplementary Fig. 2:** Figure shows lateral and top view of *SLC26A4* dimer in complex with Cl^-^ ions predicted by AlphaFold 3. Positions of variants subjected to functional assessment in this study are indicated. The effect of these mutations on protein structure is displayed on the left, showing WT (left, ConSurf grading coloring scale) and mutated (green) chains. The residues as well as H-bonds (blue dashed lines) of given WT and mutated amino acids are shown.

**References**

Abou Tayoun AN, Pesaran T, DiStefano MT, Oza A, Rehm HL, Biesecker LG, et al. Recommendations for interpreting the loss of function PVS1 ACMG/AMP variant criterion. Hum Mutat. 2018;39(11):1517-24. doi: 10.1002/humu.23626

Adler J, Parmryd I. Quantifying colocalization by correlation: the Pearson correlation coefficient is superior to the Mander's overlap coefficient. Cytometry Part A : the journal of the International Society for Analytical Cytology. 2010;77(8):733-42. doi: 10.1002/cyto.a.20896

Adzhubei IA, Schmidt S, Peshkin L, Ramensky VE, Gerasimova A, Bork P, et al. A method and server for predicting damaging missense mutations. Nat Methods. 2010;7(4):248–9. doi:10.1038/nmeth0410-248

Coyle B, Reardon W, Herbrick JA, Tsui LC, Gausden E, Lee J, et al. Molecular analysis of the PDS gene in Pendred syndrome. Hum Mol Genet. 1998;7(7):1105-12. doi: 10.1093/hmg/7.7.1105

de Moraes VCS, Bernardinelli E, Zocal N, Fernandez JA, Nofziger C, Castilho AM, et al. Reduction of Cellular Expression Levels Is a Common Feature of Functionally Affected Pendrin (SLC26A4) Protein Variants. Mol Med. 2016;22:41-53. doi: 10.2119/molmed.2015.00226

DePristo MA, Banks E, Poplin R, Garimella KV, Maguire JR, Hartl C, et al. A framework for variation discovery and genotyping using next-generation DNA sequencing data. Nat Genet. 2011;43(5):491-8. doi: 10.1038/ng.806

Dossena S, Rodighiero S, Vezzoli V, Bazzini C, Sironi C, Meyer G, et al. Fast fluorometric method for measuring pendrin (SLC26A4) Cl-/I- transport activity. Cell Physiol Biochem. 2006;18(1-3):67-74. doi: 10.1159/000095164

Dror AA, Politi Y, Shahin H, Lenz DR, Dossena S, Nofziger C, et al. Calcium oxalate stone formation in the inner ear as a result of an Slc26a4 mutation. J Biol Chem. 2010;285(28):21724-35. doi: 10.1074/jbc.M110.120188

Du Bois D, Du Bois EF. A formula to estimate the approximate surface area if height and weight be known. 1916. Nutrition. 1989;5(5):303-11; discussion 12-3.

Jaganathan K, Kyriazopoulou Panagiotopoulou S, McRae JF, Darbandi SF, Knowles D, Li YI, et al. Predicting Splicing from Primary Sequence with Deep Learning. Cell. 2019;176(3):535-48.e24. doi:10.1016/j.cell.2018.12.015

Li H, Durbin R. Fast and accurate short read alignment with Burrows-Wheeler transform. Bioinformatics. 2009;25(14):1754-60. doi: 10.1093/bioinformatics/btp324

Lofrano-Porto A, Barra GB, Nascimento PP, Costa PG, Garcia EC, Vaz RF, et al. Pendred syndrome in a large consanguineous Brazilian family caused by a homozygous mutation in the SLC26A4 gene. Arq Bras Endocrinol Metabol. 2008;52(8):1296-303. doi: 10.1590/s0004-27302008000800015

Matulevicius A, Bernardinelli E, Brownstein Z, Roesch S, Avraham KB, Dossena S. Molecular Features of SLC26A4 Common Variant p.L117F. J Clin Med. 2022;11(19). doi: 10.3390/jcm11195549

McKenna A, Hanna M, Banks E, Sivachenko A, Cibulskis K, Kernytsky A, et al. The Genome Analysis Toolkit: a MapReduce framework for analyzing next-generation DNA sequencing data. Genome Res. 2010;20(9):1297-303. doi: 10.1101/gr.107524.110

McLaren W, Gil L, Hunt SE, Riat HS, Ritchie GR, Thormann A, et al. The Ensembl Variant Effect Predictor. Genome Biol. 2016;17(1):122. doi: 10.1186/s13059-016-0974-4

Oza AM, DiStefano MT, Hemphill SE, Cushman BJ, Grant AR, Siegert RK, et al. Expert specification of the ACMG/AMP variant interpretation guidelines for genetic hearing loss. Hum Mutat. 2018;39(11):1593-613. doi: 10.1002/humu.23630

Paila U, Chapman BA, Kirchner R, Quinlan AR. GEMINI: integrative exploration of genetic variation and genome annotations. PLoS Comput Biol. 2013;9(7):e1003153. doi: 10.1371/journal.pcbi.1003153

Procino G, Milano S, Tamma G, Dossena S, Barbieri C, Nicoletti MC, et al. Co-regulated pendrin and aquaporin 5 expression and trafficking in Type-B intercalated cells under potassium depletion. Cell Physiol Biochem. 2013;32(7):184-99. doi: 10.1159/000356638

Richards S, Aziz N, Bale S, Bick D, Das S, Gastier-Foster J, et al. Standards and guidelines for the interpretation of sequence variants: a joint consensus recommendation of the American College of Medical Genetics and Genomics and the Association for Molecular Pathology. Genet Med. 2015;17(5):405-24. doi: 10.1038/gim.2015.30

Roesch S, Rasp G, Sarikas A, Dossena S. Genetic Determinants of Non-Syndromic Enlarged Vestibular Aqueduct: A Review. Audiol Res. 2021;11(3):423-42. doi: 10.3390/audiolres11030040

Sim NL, Kumar P, Hu J, Henikoff S, Schneider G, Ng PC. SIFT web server: predicting effects of amino acid substitutions on proteins. Nucleic Acids Res. 2012;40:W452–W457. doi: 10.1093/nar/gks539

Tan A, Abecasis GR, Kang HM. Unified representation of genetic variants. Bioinformatics. 2015;31(13):2202-4. doi: 10.1093/bioinformatics/btv112

Van der Auwera GA, Carneiro MO, Hartl C, Poplin R, Del Angel G, Levy-Moonshine A, et al. From FastQ data to high confidence variant calls: the Genome Analysis Toolkit best practices pipeline. Curr Protoc Bioinformatics. 2013;43(1110):11.0.1-.0.33. doi: 10.1002/0471250953.bi1110s43

Walker LC, Hoya M, Wiggins GAR, Lindy A, Vincent LM, Parsons MT, et al. Using the ACMG/AMP framework to capture evidence related to predicted and observed impact on splicing: Recommendations from the ClinGen SVI Splicing Subgroup. Am J Hum Genet. 2023;110(7):1046-67. doi: 10.1016/j.ajhg.2023.06.002

Wu CC, Lu YC, Chen PJ, Yeh PL, Su YN, Hwu WL, et al. Phenotypic analyses and mutation screening of the SLC26A4 and FOXI1 genes in 101 Taiwanese families with bilateral nonsyndromic enlarged vestibular aqueduct (DFNB4) or Pendred syndrome. Audiol Neurootol. 2010;15(1):57-66. doi: 10.1159/000231567
